# Unraveling the molecular interactions involved in phase separation of glucocorticoid receptor

**DOI:** 10.1186/s12915-020-00788-2

**Published:** 2020-06-02

**Authors:** Martin Stortz, Adali Pecci, Diego M. Presman, Valeria Levi

**Affiliations:** 1 Instituto de Química Biológica de la Facultad de Ciencias Exactas y Naturales (IQUIBICEN), CONICET-Universidad de Buenos Aires, Facultad de Ciencias Exactas y Naturales, C1428EGA Buenos Aires, Argentina; 2 Facultad de Ciencias Exactas y Naturales, Departamento de Fisiología, Biología Molecular y Celular, Universidad de Buenos Aires, C1428EGA Buenos Aires, Argentina; 3 Instituto de Fisiología, Biología Molecular y Neurociencias (IFIBYNE), CONICET-Universidad de Buenos Aires, Facultad de Ciencias Exactas y Naturales, C1428EGA Buenos Aires, Argentina; 4 Facultad de Ciencias Exactas y Naturales, Departamento de Química Biológica, Universidad de Buenos Aires, C1428EGA Buenos Aires, Argentina

**Keywords:** Glucocorticoid receptor, Membraneless organelles, Phase separation, Biomolecular condensates, Live-cell imaging, Transcription factor

## Abstract

**Background:**

Functional compartmentalization has emerged as an important factor modulating the kinetics and specificity of biochemical reactions in the nucleus, including those involved in transcriptional regulation. The glucocorticoid receptor (GR) is a ligand-activated transcription factor that translocates to the nucleus upon hormone stimulation and distributes between the nucleoplasm and membraneless compartments named nuclear foci. While a liquid-liquid phase separation process has been recently proposed to drive the formation of many nuclear compartments, the mechanisms governing the heterogeneous organization of GR in the nucleus and the functional relevance of foci formation remain elusive.

**Results:**

We dissected some of the molecular interactions involved in the formation of GR condensates and analyzed the GR structural determinants relevant to this process. We show that GR foci present properties consistent with those expected for biomolecular condensates formed by a liquid-liquid phase separation process in living human cells. Their formation requires an initial interaction of GR with certain chromatin regions at specific locations within the nucleus. Surprisingly, the intrinsically disordered region of GR is not essential for condensate formation, in contrast to many nuclear proteins that require disordered regions to phase separate, while the ligand-binding domain seems essential for that process. We finally show that GR condensates include Mediator, a protein complex involved in transcription regulation.

**Conclusions:**

We show that GR foci have properties of liquid condensates and propose that active GR molecules interact with chromatin and recruit multivalent cofactors whose interactions with additional molecules lead to the formation of a focus. The biological relevance of the interactions occurring in GR condensates supports their involvement in transcription regulation.

## Background

Recent studies have changed our view on how transcription is achieved and regulated, showing that certain properties of the intranuclear milieu are key determinants in transcriptional regulation [1–4]. The overcrowded and architecturally complex nuclear environment imposes constraints to the motion of many transcription-related molecules, critically affecting the target-searching process [5]. Additionally, many components of the transcriptional machinery, including RNA polymerases, coregulators, and transcription factors do not distribute homogeneously in the nuclear space but concentrate in membraneless domains [6–9], also affecting the probability of interaction with chromatin targets and other transcription-related molecules. These observations stress the necessity of understanding how subnuclear compartmentalization is established and its impact on transcription.

More generally, compartmentalization may modulate the kinetics [10] and specificity [11] of biochemical reactions by locally increasing or reducing the concentrations of specific reactants with respect to those in the nucleoplasm [12, 13]. Also, these domains may act as storage centers, sequestering certain biomolecules and/or buffering their concentration in the nucleoplasm [12–15]. In addition, some domains may function as hubs for chromatin organization [13, 16, 17].

The glucocorticoid receptor (GR) is a ligand-regulated transcription factor involved in a plethora of physiological functions ranging from metabolism to the immune system response [18]. Upon ligand activation, the receptor translocates to the nucleus where it distributes between the nucleoplasm and numerous foci [19]. Foci formation was verified for the endogenous GR by immunocytochemistry [19] and for the receptor fused to a fluorescent protein in transient expression assays [20–22]. Despite this heterogeneous distribution is common among other steroid receptors [23–26], its biological function still remains controversial. Whereas foci are not considered as active transcription domains [19, 27], some observations suggest that they may have an indirect impact on transcription. Particularly, foci formation depends on the receptor conformation [20, 21, 28] and it also involves a redistribution of other GR-associated factors such as the GR coactivator NCoA-2/SRC-2 which initially concentrates in PML bodies and re-localizes to GR foci upon receptor activation [20].

Recently, it was proposed that a liquid-liquid phase separation process drives the formation of many membraneless compartments in living cells, producing a paradigm shift in the cell biology field [11–13, 29–32]. Under this new model, the nucleus emerges as a multi-phase compartment and the nuclear biomolecules undergo a dynamic, modulated partition between these different liquid phases. This paradigm, which is a matter of active debate [33, 34], provides a new framework to understand how membraneless organelles are formed and maintained in cells [35]. Phase separation seems to play an important role in the control of many nuclear processes, including gene expression regulation [36–38].

In this work, we address whether GR foci form as a consequence of a liquid-liquid phase separation process. We used a combination of chemical treatments and GR mutants followed by live-cell imaging to characterize the biophysical properties of GR foci and to explore the molecular interactions involved in foci formation. Our results suggest that GR foci formation requires an initial interaction with certain chromatin regions followed by a liquid-liquid phase separation process with a potentially relevant functional role in the transcriptional activity of the glucocorticoid receptor.

## Results

### GR foci exhibit physicochemical features consistent with liquid condensates

**Fig. 1:**
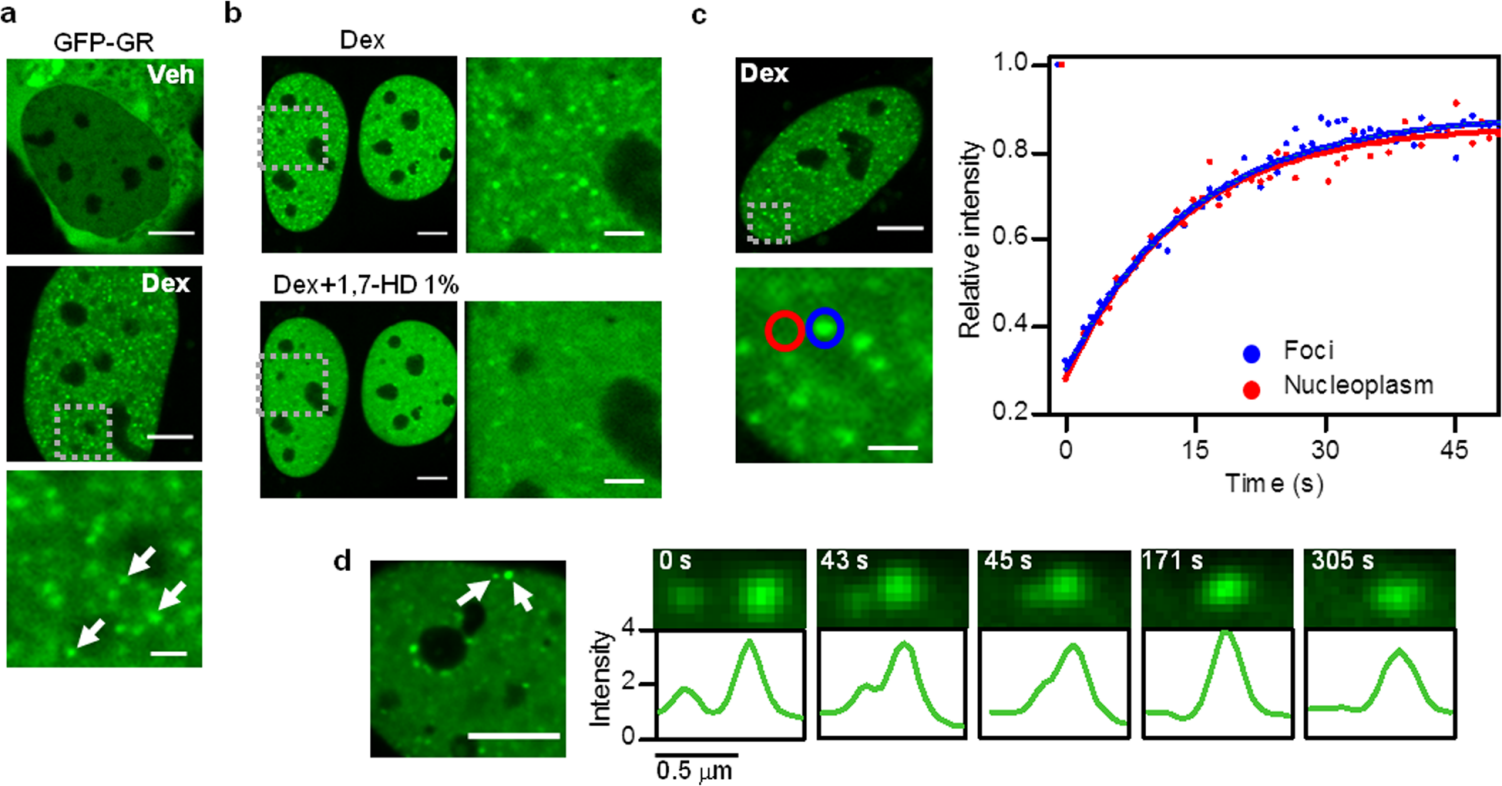
**GR foci behave as phase-separated condensates.** U2OS cells expressing GFP-GR were incubated with Dex and imaged by confocal microscopy. **a** Representative images of cells incubated with vehicle (Veh) or Dex (Scale bar: 5 μm). The zoomed image corresponds to the region indicated by the dashed square (Scale bar: 1 μm). The white arrows point to some GR foci. See also Additional file 1: Supplementary Video S1. **b** Representative images of the same cells before and after a treatment with 1% *v*/*v* 1,7-heptanediol (1,7-HD) for 30 s (Scale bar: 5 μm). The zoomed images correspond to the regions indicated by the dashed squares (Scale bar: 2 μm). This experiment was run in 20 cells obtaining similar results. **c** Representative results obtained in FRAP experiments in which the intensity recovery in adjacent regions corresponding to a GR focus (blue circle) and the nucleoplasm (red circle) were analyzed (Scale bar: 5 μm). The zoomed image corresponds to the region indicated by the dashed square (Scale bar: 1 μm). Fluorescence intensity was doubly normalized to the intensity of the same region before bleaching and to the intensity of a non-bleached reference region at the same time point. Time = 0 s corresponds to the beginning of the image sequence showing the fluorescence recovery. Data was fitted considering a simple exponential model (Eq. 2). Fits are shown with a continuous blue (foci) and red (nucleoplasm) lines. Raw data can be found in Additional file 13: Supplementary Table S1. **d** Time-lapse imaging showing a fusion event between two GR foci, indicated with white arrows before fusion (Scale bar: 5 μm). Fluorescence intensity profiles along the foci at different time points of the image stack. The intensity is represented relative to that determined in the nucleoplasm. Five fusion events were detected in 48 time-lapse sequences of different cells. See also Additional file 5: Supplementary Video S2


To visualize GR foci formation in living cells, we stimulated human U2OS cells transiently expressing GR fused to GFP (GFP-GR) with the synthetic agonist dexamethasone (Dex). Upon ligand stimulation, GR translocated to the nucleus and formed foci within a few minutes (Fig. 1a and Additional file 1: Supplementary Video S1). The mean foci density determined at the central *z*-plane of nuclei was 0.54 + 0.02 foci/μm^2^ (*n*_cells_ = 146). Additional file 2: Supplementary Figure S1 shows that the size of these structures is below or near the optical resolution limit (~ 230 nm, [39]). We first assessed if key physicochemical properties of these GR foci are compatible to those expected for liquid condensates. We incubated the cells with 1,7-heptanediol (1,7-HD) (1%v/v) since a very similar aliphatic alcohol (1,6-hexanediol) disrupts the weak intermolecular interactions that usually stabilize biomolecular condensates and thus it has been used to identify liquid condensates [40, 41]. This treatment promoted a partial disassembly of GR foci after a few seconds of its addition (Fig. 1b). Indeed, we determined the foci density at a single-cell level before and after the treatment and verified that the foci density was reduced to 36.8 + 4.9% after 1,7-HD addition. We tested higher 1,7-HD concentrations (3–10%v/v)—similar to those previously used in the literature for 1,6-hexanediol [9, 36, 42]—and confirmed that even though they decreased the foci density, the integrity of the cells was severely compromised (Additional file 3: Supplementary Fig. S2).

**Additional file 1:** Supplementary **Video S1.** Related to Fig. 1. GR subcellular dynamics upon hormone stimulation. U2OS cells expressing GFP-GR were incubated with Dex and time-lapsed imaged by confocal microscopy for 19 min using a frame time of 1 min. The images stack initiates with the Dex addition (Scale bar: 5 μm).


We also analyzed the effects of 1,7-HD in mouse mammary adenocarcinoma 3617 cells. This cell line [43] carries a GFP-GR transgene under the control of the Tet-off system, leading to GFP-GR levels similar to those of the endogenously expressed GR [44]. Additional file 4: Supplementary Figure S3a shows that the density of GR foci in 3617 cells also decreased after 1,7-HD treatment ruling out the fact that foci sensitivity to 1,7-HD is due to GR overexpression.

Additionally, we checked that the intensity levels of 3617 cells stably expressing GFP-GR are similar to those of the U2OS cells transiently expressing GFP-GR that meet the criterion described in “Materials and methods” for imaging experiments (Additional file 4: Supplementary Fig. S3b). This result shows that the U2OS cells analyzed in this work express GR-GFP levels similar to the endogenous concentration of the receptor ruling out the possibility of artifacts due to transient transfection. The sensitivity of GR foci to 1,7-HD suggests that weak hydrophobic interactions are involved in their formation and/or stabilization, consistent with the liquid-liquid phase separation model.

Biomolecular condensates are dynamic structures that present fast exchange of molecules with their surroundings [45]. Consistently with this property, fluorescence recovery after photobleaching (FRAP) experiments at foci and nearby regions of the nucleoplasm revealed similar recovery dynamics (Fig. 1c). We fitted FRAP data with an empirical exponential equation to calculate a characteristic recovery time scale (*τ*_c_) and we estimated a mean *τ*_c,foci_/*τ*_c,nucleoplasm_ ratio of 1.00 + 0.09 (*n*_cells_ = 6). This result agrees with previous fluorescence correlation spectroscopy data showing a fast exchange of GR molecules at foci [20].

Another hallmark of liquid condensates is the coalescence of separated droplets that merge into larger ones thus minimizing surface tension [29, 46–48]. Despite the reduced mobility of foci in the nucleus, we could detect some of these fusion events (Fig. 1d and Additional file 5: Supplementary Video S2). The fused foci persisted for long periods of time and moved as a single entity, suggesting that these events are not an artifact of observing foci located at different *z*-planes. This result also supports a liquid-liquid phase separation of GR foci.


**Additional file 5:** Supplementary **Video S2.** Related to Fig. 1. Fusion between two GR condensates. U2OS cells expressing GFP-GR were incubated with Dex for 30 min and time-lapsed imaged by confocal microscopy for 2.8 min using a frame time of 0.331 s (Image size: 2 μm).


**Fig. 2:**
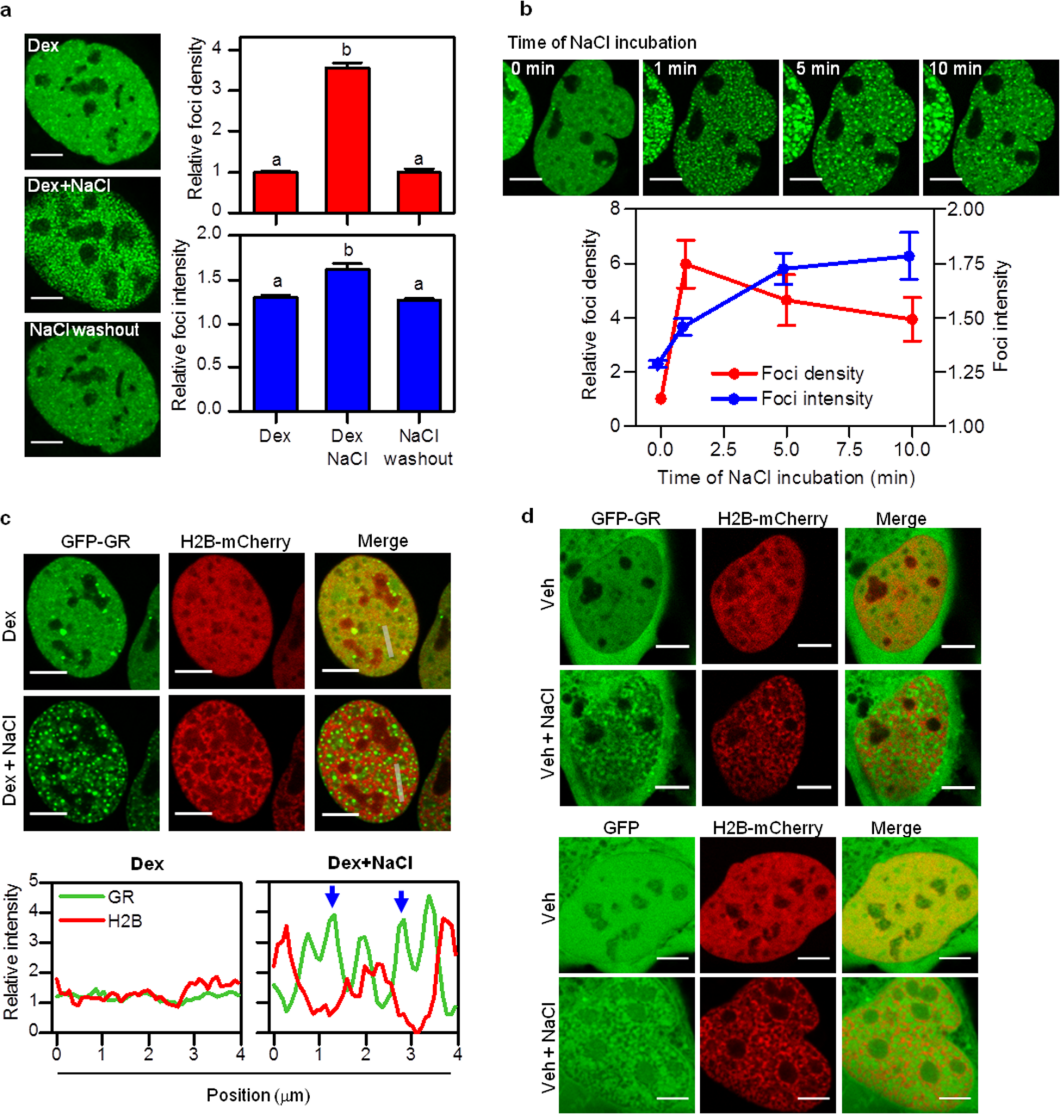
**GR condensates are modulated by osmolarity changes. a**, **b** U2OS cells expressing GFP-GR were incubated with Dex, then with medium supplemented with 100 mM NaCl for 1 min and imaged by confocal microscopy (Scale bar: 5 μm). **a** Representative images of the same cell before and after NaCl incubation, and after re-introducing the cells in isotonic medium (NaCl washout). (Top panel) Mean foci density relative to the density obtained before NaCl incubation in the same cells. (Bottom panel) Mean foci intensity relative to the intensity of the nucleus in each image. Bars with different superscript letters indicate data significantly different (*p* < 0.05) (*n*_cells_ = 5). Raw data can be found in Additional file 14: Supplementary Table S2. **b** Representative images of the same cell before and after incubation with NaCl at the indicated time points. Mean foci density (red line) and mean foci intensity (blue line) (*n*_cells_ = 4). Foci intensity relative to the intensity of the nucleus in each image. Foci density relative to the density before NaCl incubation. Raw data can be found in Additional file 14: Supplementary Table S2. **c** U2OS cells co-expressing GFP-GR and H2B-mCherry were incubated with Dex, then with medium supplemented with 100 mM NaCl for 5 min and imaged by confocal microscopy. Representative images of the same cells before and after with NaCl (Scale bar: 5 μm). Intensity profiles obtained along the lines indicated with gray lines in the merged images. The intensity is represented relative to that determined in the nucleoplasm. Blue arrows point to the position of GR hyperosmotic-induced dots (HOIDs), which are excluded from chromatin condensates. This experiment was run in 12 cells obtaining a similar anticolocalization pattern. **d** U2OS cells co-expressing H2B-mCherry and GFP-GR or GFP were incubated with vehicle (Veh), then with medium supplemented with 100 mM NaCl for 1 min and imaged by confocal microscopy. Representative images of cells before and after NaCl incubation (Scale bar: 5 μm). This experiment was run in six cells for each condition obtaining similar distributions

We next tested if GR foci respond to osmolarity changes as liquid-liquid phase separation depends on several physicochemical properties of the medium [13, 49–52]. Incubating the cells with a hyperosmotic medium (culture medium supplemented with 100 mM NaCl) triggered a fast and massive reorganization of foci that includes the formation of a higher number of more intense dots (“hyperosmotic-induced dots,” HOIDs) (Fig. 2a). The addition of NaCl also induced the formation of HOIDs in 3617 cells stably expressing endogenous levels of GFP-GR (Additional file 4: Supplementary Fig. S3c). After a few minutes of incubation with hyperosmotic medium, GR HOIDs coalesced to a lower number of even brighter and larger HOIDs (Fig. 2b). GR HOIDs disassembled after restoring the medium to isotonic conditions (Fig. 2a), indicating that these structures are not irreversible aggregates. Indeed, we did not find statistical differences (*p* = 0.67) between the mean foci densities in cells in isotonic medium (relative foci density = 1.00 + 0.05; *n* = 26) and in cells that were incubated in hyperosmotic medium and restored to isotonic medium afterwards (relative foci density = 0.97 + 0.05; *n* = 25). Noticeably, we observed similar effects when we increased the osmolarity of the medium with sucrose (Additional file 6: Supplementary Fig. S4a), suggesting that the loss of cellular water may be responsible for these changes. Consistent with this hypothesis, the hypertonic shock reduced ~ 30% the nuclear volume which in turn resulted in a ~ 55% increase in GR nuclear concentration (Additional file 6: Supplementary Fig. S4b). The hypertonic treatment also triggered a drastic chromatin condensation as revealed by the intensity distribution of H2B (Fig. 2c). Interestingly, these hyperosmotic-induced condensed chromatin regions excluded GR HOIDs (Fig. 2c).

To test if HOIDs were a consequence of an unspecific aggregation in hyperosmotic medium, we also assayed the effects of the hypertonic treatment in cells expressing GFP or the inactive GFP-GR (i.e., without Dex stimulation). These proteins were excluded from dense chromatin regions resulting in relatively extended and dimmer domains with irregular shapes different from the droplet-like HOIDs formed by the active receptor (Fig. 2d and Additional file 6: Supplementary Fig. S4c). These results suggest that HOID formation requires specific interactions involving the active GR.

Taken together, the disruption of GR foci with 1,7-HD, the ability of foci to coalesce, the fast dynamics of GR molecules in these domains, and the sensitivity of foci to osmolarity changes suggest that GR foci present some physical characteristics of biomolecular condensates generated by liquid-liquid phase separation. In the following sections, we will present other results that also support this hypothesis.

### GR foci formation requires association with chromatin

**Fig. 3:**
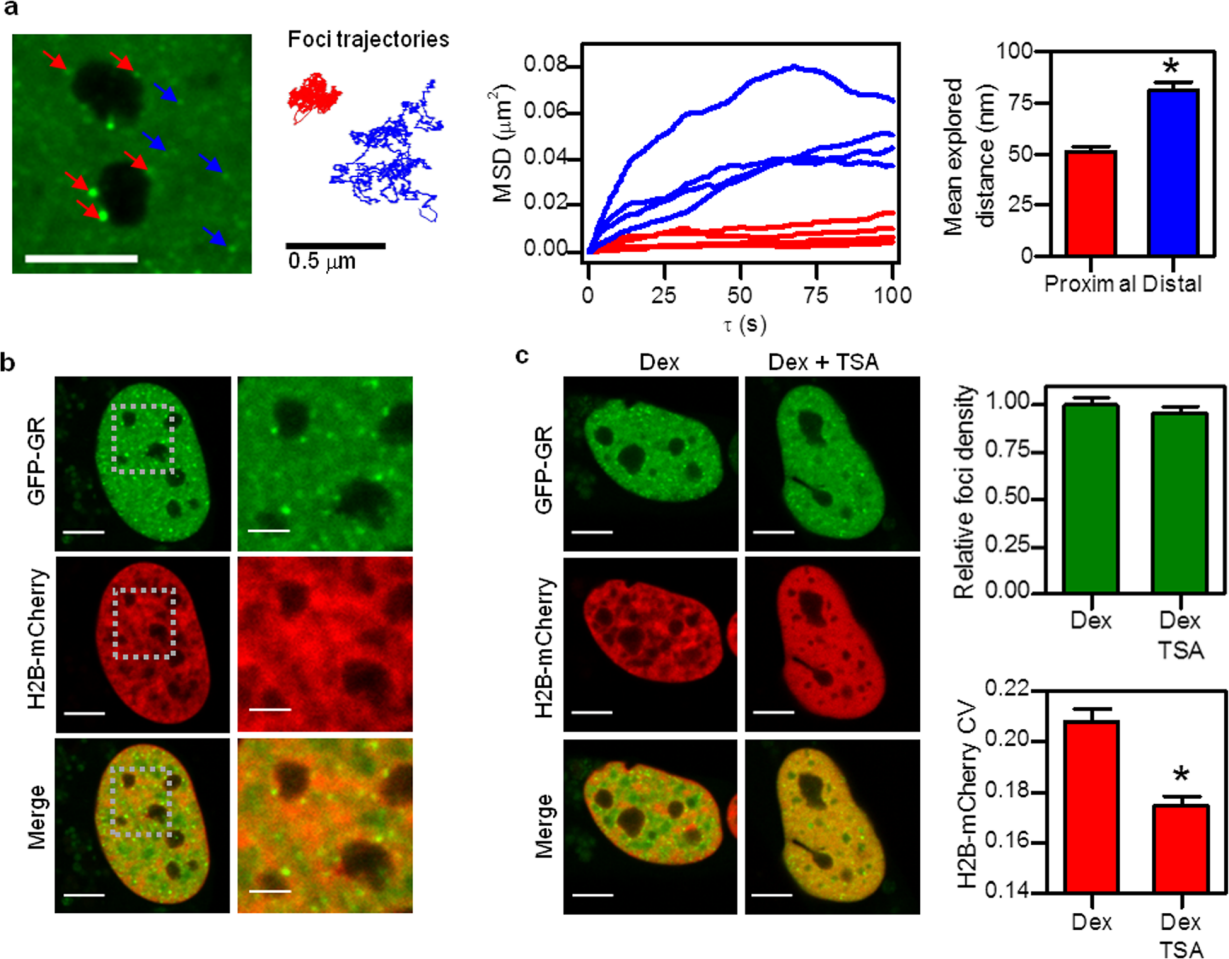
**Relation between the formation of GR condensates and chromatin. a** U2OS cells expressing GFP-GR were incubated with Dex and time-lapse imaged by confocal microscopy. 2D trajectories of GR foci were obtained by single-particle tracking. Red arrows point to foci very close to nucleoli, blue arrows show foci far from these structures (Scale bar: 5 μm). (Left panel) Representative trajectories obtained from these analyses. (Middle panel) The mean square displacement (MSD) was calculated as a function of the time lag (*τ*) according to Eq. 3. MSD(*τ*) data obtained for representative trajectories of foci close to nucleoli (red, proximal) or far from them (blue, distal). (Right panel) The mean explored distance (MED_*τ* = 100_) was calculated from the MSD values at *τ* = 100 s. An average MED_*τ* = 100_ was calculated for the proximal and distal populations of foci. The bar marked with an asterisk (*) represents data significantly different (*p* < 0.05) (Proximal: *n*_foci_ = 30; Distal: *n*_foci_ = 50; *n*_cells_ = 7). Raw data can be found in Additional file 15: Supplementary Table S3. See also Additional file 7: Supplementary Video S3. **b**, **c** U2OS cells co-expressing GFP-GR and H2B-mCherry were incubated with Dex and imaged by confocal microscopy (Scale bar: 5 μm). **b** The zoomed image corresponds to the region indicated by the dashed square (Scale bar: 2 μm). **c** U2OS cells were incubated 16 h with TSA 1 μg/ml before Dex stimulation (Top panel) GR foci density relative to the mean density of cells only treated with Dex (Dex: *n*_cells_ = 82; Dex + TSA: *n*_cells_ = 71). (Bottom panel) Coefficient of variation (CV) of H2B-mCherry intensity in the nucleus (Dex: *n*_cells_ = 25; Dex + TSA: *n*_cells_ = 18). The bar marked with an asterisk (*) represents data significantly different (*p* < 0.05) from cells only treated with Dex. Raw data can be found in Additional file 15: Supplementary Table S3


Liquid condensates usually involve multivalent scaffolds such as chromatin or RNA that act as nucleation centers for the phase separation of recruited client molecules [12, 13, 48]. Previously, we have reported that in situ DNase I digestion disrupts GR foci organization [20], thus suggesting that DNA is likely involved in foci formation and may even constitute the scaffold for foci establishment. To further characterize the dynamical organization of GR condensates, we run single-particle tracking experiments and recovered 2D trajectories of foci with nanometer precision (Fig. 3a and Additional file 7: Supplementary Video S3). We next examined these trajectories through a mean square displacement (MSD) analysis since it provides details on the mechanisms underlying their motion [53].

**Additional file 7:** Supplementary **Video S3.** Related to Fig. 3. 2D motion of GR foci. U2OS cells expressing GFP-GR were incubated with Dex for 30 min and time-lapsed imaged by confocal microscopy for 5.5 min using a frame time of 0.331 s (Scale bar: 2 μm).


Most foci explored a very small area in the assayed time window (100 s) (Fig. 3a), within the order observed for DNA-bound histones by single-molecule tracking (~ 250–300 nm, [54]). This observation is consistent with GR liquid condensates constrained by and/or associated to the chromatin network. We identified at least two populations of foci with different motility properties. The first population, close to nucleoli (“proximal foci,” distance to nucleoli < 0.5 μm), presented highly constrained dynamics (Fig. 3a, red) while the second, located at larger distances (“distal foci”), explored relatively larger nuclear regions (Fig. 3a, blue). Proximal foci seem to incorporate more GR molecules than distal foci but their mean explored distance does not depend on their relative intensity (Additional file 8: Supplementary Fig. S5), supporting that the motion of these structures is constrained by interactions with a large, relatively immobile structure (as expected from an indirect/direct association with nucleoli). On the other hand, distal and brighter foci explored a smaller area in comparison to distal, dimmer foci (Additional file 8: Supplementary Fig. S5). This result suggests that distal foci may be linked to less-restricted chromatin and their motion probably depends on both, the interaction with chromatin and the number of GR molecules per focus. We should mention that those condensates that incorporate more GR molecules are probably bigger, and thus, they move slower.

These results suggest that foci may form in close association with specific nuclear compartments, e.g., nucleoli, or at other less restricted regions of the nuclear space.

To better examine the role of chromatin in the formation of GR condensates, we first analyzed if there is a correlation between foci formation and the local condensation of chromatin (Fig. 3b). We localized GFP-GR foci as described in “Materials and methods,” determined the H2B-mCherry signal intensity (related to the chromatin compaction state [55]) at the foci positions, and compared this value with the mean nuclear intensity (Int_H2B,foci_/Int_H2B*,*nucleus_ ratio). This ratio is 0.999 + 0.005 (*n*_foci_ = 1183; *n*_cells_ = 13), suggesting that GR condensates do not require a certain chromatin compaction state for their formation. In line with this observation, trichostatin A (TSA), a histone deacetylase inhibitor that produces a global, dramatic chromatin decompaction [56], did not affect GR foci formation (Fig. 3c).

**Fig. 4:**
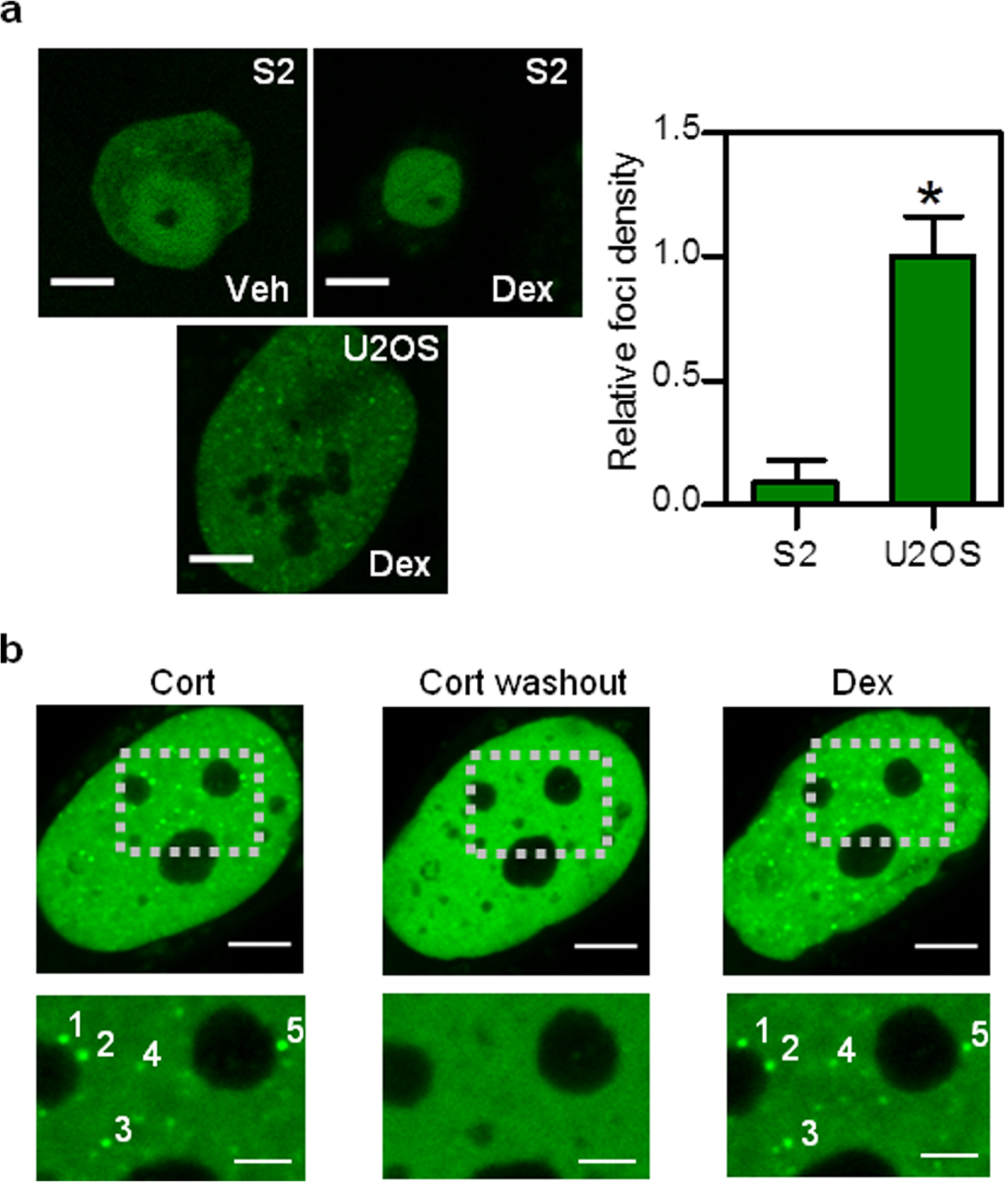
**GR condensates formation requires interactions with specific chromatin regions. a** Representative images of *Drosophila melanogaster* S2 and U2OS cells expressing GFP-GR incubated with vehicle (Veh) or Dex and imaged by confocal microscopy with the same microscope settings (Scale bar: 5 μm). The asterisk (*) denotes a significantly different foci density (*p* < 0.05) with respect to that obtained for S2 cells (S2: *n*_cells_ = 8; U2OS: *n*_cells_ = 13). Raw data can be found in Additional file 16: Supplementary Table S4. **b** U2OS cells expressing GFP-GR were incubated with 100 mM corticosterone (Cort) for 1 h, then washed with PBS five times for 15 min (Cort washout) and finally incubated with Dex for 15 min. Representative images of a cell in the three conditions (Scale bar: 5 μm). The zoomed images correspond to the regions indicated by the dashed squares (Scale bar: 2 μm). Foci that present close positions in Cort and Dex conditions are indicated with the same number (1–5). This experiment was run in seven cells obtaining similar results

We next assayed if foci formation only requires active GR molecules (i.e., foci result from a GR self-association process that is independent of the cellular context) and tested if the active GR forms condensates in *Drosophila melanogaster* S2 cells. *Drosophila* is an excellent model as it does not express any known GR orthologue [57]. In addition, its nuclear receptors keep little sequence identity with the GR and they bind to different DNA sequences [57–59]. Moreover, ectopically GR expression can induce a transient glucocorticoid response element-driven reporter gene in *Drosophila* cells [60]. We transiently expressed GFP-GR in S2 cells and observed that the receptor translocated from the cytoplasm to the nucleus upon Dex stimulus (Fig. 4a). Noticeably, the Dex-activated GR failed to form foci in the nucleus of S2 cells (Fig. 4a). We ruled out that the low foci number was a consequence of lower expression levels of GFP-GR in this cell line as U2OS cells expressing similar levels of GFP-GR present distinguishable foci (Fig. 4a). Therefore, the inability of the receptor to form foci in these non-mammalian cells indicates that GR in its active conformation is not sufficient to form foci and thus GR foci formation does not rely on a simple self-association of the receptor. Overall, our data suggests that GR condensates include certain cofactors and/or chromatin regions that are absent in *Drosophila* cells.

Chromosomes and gene loci occupy defined positions within the nucleus [6]. Thus, we hypothesized that GR condensates could form at specific positions in the nucleus if their formation requires the recruitment of active GR molecules to specific chromatin regions. To test this hypothesis, we analyzed in single cells if the positions of foci are maintained after removing the glucocorticoid ligand and stimulating the cells again (Fig. 4b). In these experiments, we stimulated the cells with the natural glucocorticoid ligand corticosterone (Cort), washed the cells to remove the ligand and re-stimulated the cells with Dex. We did not use Dex in the first stimulation since the small dissociation constant of the GR-Dex complex limits the possibility to rapidly reverse GR activation [61]. Strikingly, many GR condensates formed after Cort and Dex stimulations assembled at similar positions in the nucleus (Fig. 4b), supporting that GR foci do not form at random positions but rather require specific interactions with certain chromatinic regions probably acting as nucleation centers.

### Mediator is part of GR foci

We have previously reported that relevant biomolecules involved in GR transcriptional control such as the coactivator NCoA-2 are recruited to GR foci [20]. Thus, we decided to test if other transcriptional coregulators are also incorporated in these condensates. Particularly, Mediator is a multi-subunit complex involved in transcription regulation [62–65] that forms liquid condensates in association with chromatin, coactivators, RNA polymerase II, and transcription factors. Since it has been demonstrated that Mediator condensates are relevant for transcriptional control [9, 66, 67], we tested if Mediator is also included in GR foci.

**Fig. 5:**
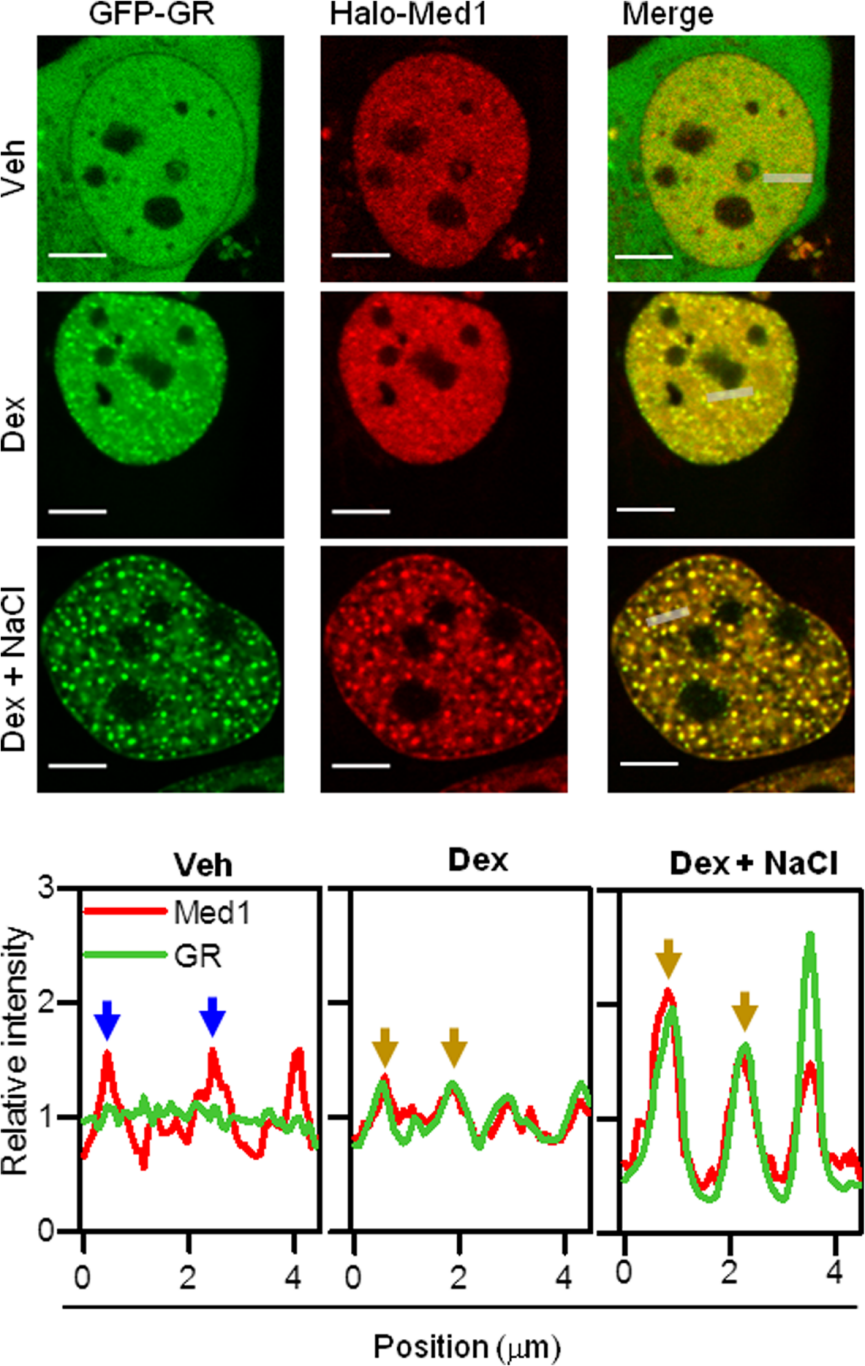
**GR condensates include Mediator.** Representative images of U2OS cells co-expressing GFP-GR and JF549-labeled Halo-Med1 treated with vehicle (Veh) or Dex, incubated with medium supplemented with 100 mM NaCl when indicated and imaged by confocal microscopy (Scale bar: 5 μm). Intensity profiles obtained along the lines indicated with gray lines in merged images. The intensity is represented relative to that determined in the nucleoplasm in each case. Blue and yellow arrows point to Med1 and Med1-GR condensates, respectively. These analyses were performed in 15 (Veh), 25 (Dex), and 5 cells (Dex + NaCl) obtaining similar distributions for each condition

We analyzed the distributions of GFP-GR and the Mediator subunit Med1 fused to HaloTag [68] and labeled with JF549 [69] in U2OS cells. Med1 concentrates in subnuclear puncta in untreated cells whereas GR distributes homogeneously, as expected for the inactive receptor (Fig. 5). Dex stimulation resulted in the formation of many foci containing both GR and Med1 (Fig. 5 and Additional file 9: Supplementary Fig. S6). On the other hand, the hypertonic treatment of these cells promoted the formation of HOIDs containing GR and Med1 as described before for GR foci (cf. Figs. 2a and 5).

Taken together, these results suggest that active GR molecules coexist at foci with Mediator, a protein also involved in the formation of liquid, transcriptional condensates.

### GR structural determinants of foci formation

GR is a modular transcription factor, organized into three structural and functional domains: the N-terminal domain (NTD), the central DNA-binding domain (DBD), and the C-terminal ligand-binding domain (LBD) [70]. To explore the role of different structural features of the receptor in the liquid-liquid phase separation process, we expressed certain GR mutants [44] in cells and analyzed their ability to form condensates.

**Fig. 6:**
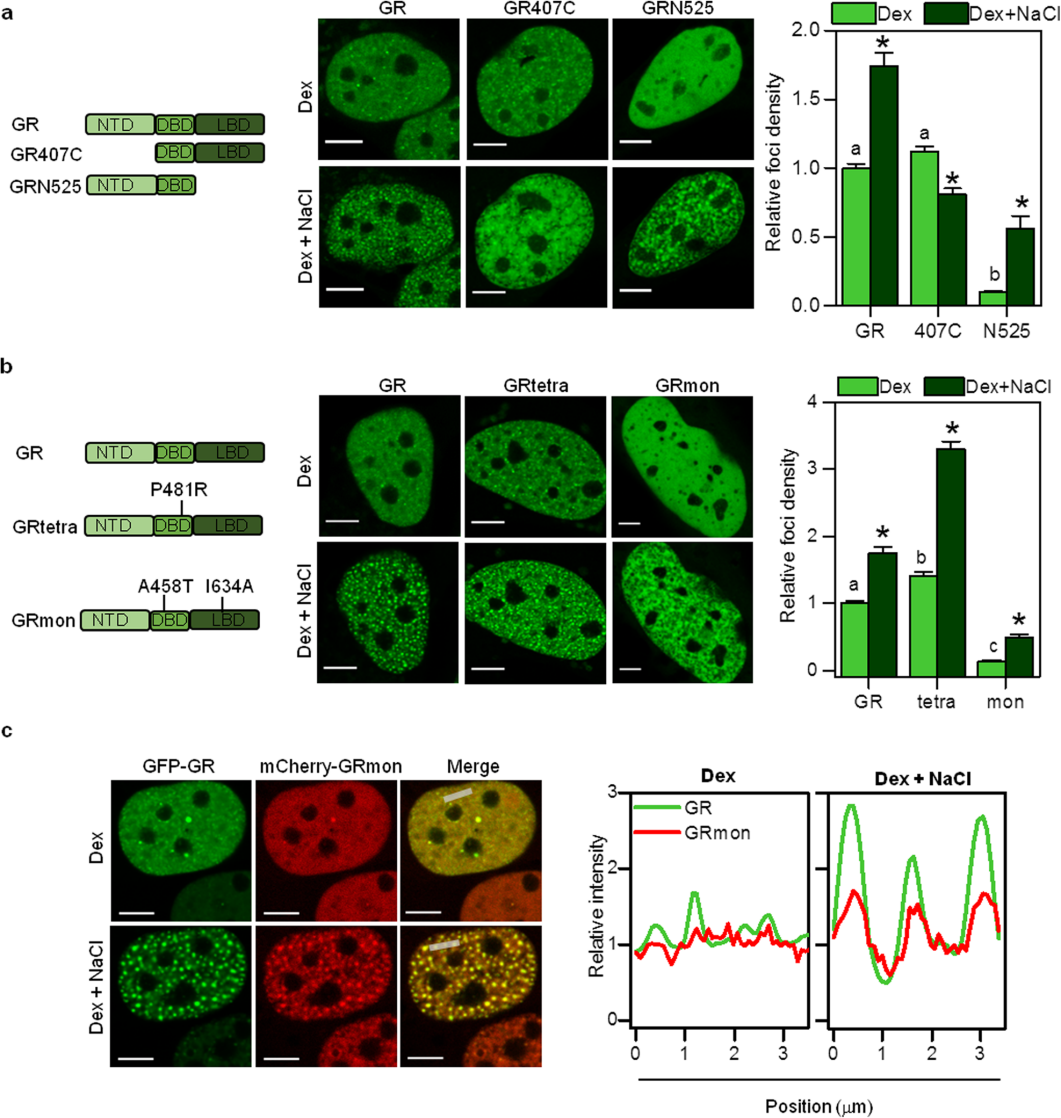
**Structural determinants involved in GR condensates formation. a**, **b** Representative images of U2OS cells expressing GFP fused to wild-type GR or mutant variants of the receptor (GR407C, GRN525, GRtetra, GRmon) incubated with Dex, before (top panels) and after incubation with medium supplemented with 100 mM NaCl (bottom panels) (Scale bar: 5 μm). The ability of the different mutants to form foci in isotonic (light green) and hypertonic (dark green) conditions was evaluated by calculating the foci density (*n*_cells,GR + Dex_ = 146; *n*_cells,GR + Dex + NaCl_ = 15; *n*_cells,407C + Dex_ = 43; *n*_cells,407C + Dex + NaCl_ = 24; *n*_cells,N525 + Dex_ = 27; *n*_cells,N525 + Dex + NaCl_ = 16; *n*_cells,tetra + Dex_ = 21; *n*_cells,tetra + Dex + NaCl_ = 12; *n*_cells,mon + Dex_ = 24; *n*_cells,mon + Dex + NaCl_ = 18). Bars with different superscript letters represent data significantly different (*p* < 0.05). The asterisk (*) denotes a significantly different foci density (*p* < 0.05) with respect to that obtained for the same GR variant in isotonic medium. Raw data can be found in Additional file 17: Supplementary Table S5. **c** Representative images of U2OS cells co-expressing GFP-GR and mCherry-GRmon incubated with Dex and then with medium supplemented with 100 mM NaCl when indicated (Scale bar: 5 μm). Intensity profiles obtained along the gray lines indicated in the merged images. The intensity is represented relative to that determined in the nucleoplasm in each case. Similar results were obtained with U2OS cells coexpressing GFP-GRmon and mCherry-GR (*n*_cells,Dex_ = 10; *n*_cells,Dex + NaCl_ = 13, including both GFP-GR + mCherry-GRmon and GFP-GRmon + mCherry-GR experiments)

Intrinsically disordered regions (IDRs) of proteins play a relevant role in the weak hydrophobic interactions required for phase separation [13, 42, 71, 72]. Since the GR presents a disordered region in its NTD [70], we analyzed the nuclear distribution of a GR mutant lacking the NTD and fused to GFP (GR407C) [44]. While this mutant forms a similar number of foci as the wild-type receptor upon Dex stimulation, the hypertonic treatment affects GR407C condensates differently (Fig. 6a). In contrast to the wild-type receptor, foci density did not increase after NaCl treatment, suggesting that the GR NTD probably plays a role in establishing interactions that may stabilize these liquid condensates. Notably, GR407C foci respond similarly to 1,7-HD treatment as the full-length GR (Additional file 10: Supplementary Fig. S7), suggesting that foci sensitivity to 1,7-HD does not rely on interactions involving GR IDR. Taken together, these results suggest that the IDR of GR plays a role in stabilization, but it is not essential for foci formation.

On the other hand, we found that the removal of the LBD (GRN525 mutant), which includes regions involved in interactions with GR transcriptional cofactors [73], drastically affected foci formation (Fig. 6a). Additionally, the GRN525 mutant distributed in a heterogeneous pattern under hypertonic conditions closely resembling the distributions of GFP and the inactive receptor (Fig. 6a). Therefore, GR LBD is essential for the formation of liquid condensates.

In a previous work, we showed that the mostly monomeric receptor GRmon (A465T/I634A) [74] does not form foci in BHK cells [20]. Also, this mutant is severely impaired in specific chromatin binding [74]. The inability of GRmon to form foci was also observed in U2OS cells (Fig. 6b) suggesting a link between foci formation and either chromatin binding, quaternary structure of the receptor, or both. In contrast to the monomeric receptor, we found that a constitutively tetrameric GR mutant (GR^P481R^, named GRtetra) that presents higher transcriptional activity and specific DNA binding in comparison to the wild-type receptor [44, 75] also shows a higher capability to form condensates (Fig. 6b). Its higher DNA binding capability and/or its possibility to establish higher valency interactions with the factors involved in condensate formation could explain the enhanced phase separation of this tetrameric mutant.

Additionally, we verified that GRmon did not form HOIDs after hypertonic treatment but distributed in an irregular, chromatin-excluded pattern similar to the GRN525 mutant (Fig. 6b, central panel). On the other hand, GRtretra phase separation increased under hypertonic conditions, forming HOIDs as the wild-type receptor (Fig. 6b). These and the overall results obtained with the different receptor mutants suggest that the pre-existence of receptor condensates is necessary for HOIDs formation.

We also quantified the intensity of foci relative to the nuclear intensity for the different mutants to analyze possible changes in the number of fluorescent molecules forming these structures. Additional file 11: Supplementary Figure S8 shows that those mutants that form fewer foci (GRmon and GRN525, Fig. 6a, b) produced dimmer structures indicating that they incorporate a lower number of fluorescent molecules. On the other hand, the wild-type receptor and the GR407C mutant presented very similar foci intensity distributions whereas the mean intensity of GRtetra foci was slightly higher (~ 2%, *p* = 0.035, Student’s *t* test) than that of foci formed by the wild-type receptor. Thus, we could hypothesize that GRtetra allows seeding condensates at a larger number of nuclear positions in comparison to the wild-type GR (Fig. 6a) but the number of molecules included in GRtetra condensates seems to be only slightly higher than those observed for the wild-type receptor.

We finally asked if the failure of GRmon mutant to form condensates is only due to its defective binding to chromatin. Co-expression of GFP-GR and mCherry-GRmon did not rescue the GRmon phenotype, as only a few brighter GR foci exhibited a local accumulation of GRmon (Fig. 6c and Additional file 12: Supplementary Fig. S9). This result shows that GRmon fails to be recruited to already formed condensates, suggesting that this mutant cannot establish the network of interactions needed to incorporate to these liquid condensates. These interactions may involve cofactors and/or other GR molecules, in addition to DNA. Interestingly, GRmon was recruited to GR HOIDs after the hypertonic treatment (Fig. 6c), even in conditions wherein this mutant could not form HOIDs by itself (Fig. 5b). This result supports the idea that hypertonic conditions favor phase separation of receptor molecules into already formed GR condensates.

## Discussion

The eukaryotic cell nucleus houses a plethora of complex biological processes occurring in a heterogeneous environment that includes a variety of membraneless compartments [6, 76]. Particularly, several biomolecules involved in transcription concentrate in nuclear sub-compartments [37, 77]. Thus, understanding the molecular principles governing the heterogeneous distribution of transcription-related molecules within the nuclear space will help us comprehend how this process is achieved and regulated in cells [2, 5]. Nuclear subdomains were considered as large and stable macromolecular assemblies governed by structured interactions between well-folded components. In recent years, this idea has been challenged by the proposition of liquid-liquid phase separation as a general mechanism driving the formation of membraneless domains [35]. Nevertheless, there is an open debate regarding the experimental evidence required to demonstrate the existence of liquid-liquid phase separation in living cells [33, 34].

**Fig. 7:**
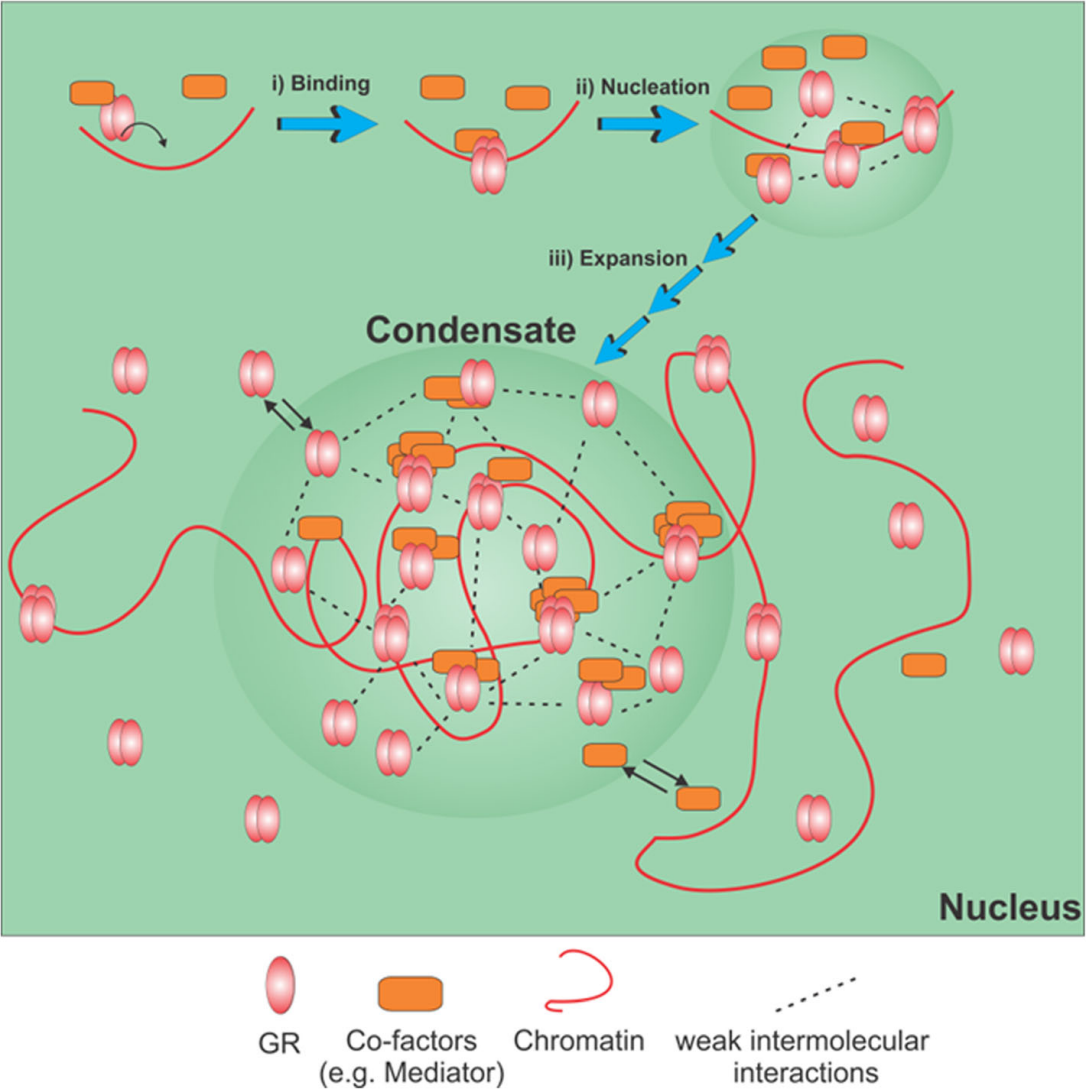
**Model of GR condensate formation.** The cartoon represents the successive steps of a GR focus formation, according to the proposed model. (i) Initially, GR directly or indirectly bind certain chromatin regions. (ii) Mediator and other multivalent cofactors form an incipient interactions network with other chromatin-bound GR molecules. (iii) The focus expands by the incorporation of a growing number of molecules through a weak interactions network. Molecules dynamically exchange from and to the focus

The distribution of active steroid receptors in numerous foci in the nucleus was observed several years ago, but the nature and role of these focal domains is far from understood [20, 78]. Here, we demonstrated that GR foci exhibit several hallmark properties of liquid condensates and explored the molecular interactions required for the GR phase separation in living cells. These findings lead us to propose a model explaining the formation of GR hubs (Fig. 7).

The initial step of GR condensate formation is the generation of a relatively structured seed that includes the active GR directly or indirectly bound to certain chromatin regions (Fig. 7, i). In support, we found that (I) GR foci are sensitive to DNase treatment [20]; (II) GR foci formation seems to require some components of the specific biochemical background of mammalian cells since GR did not phase separate in the nucleus of *Drosophila* cells (Fig. 4a); (III) foci do not produce at random positions of the nucleus but at specific regions (Fig. 4b); and (IV) the GRtetra mutant has an enhanced ability to form foci, whereas the GRmon mutant, with impaired DNA binding, does not phase separate (Fig. 6b). In line with this proposition, a current model explaining the formation of transcriptional condensates [79] places DNA as a multivalent scaffold that specifically recruit transcription factors through high affinity, structured interactions with their DNA-binding domains.

In a subsequent step, the initial seed recruits GR and likely other partner molecules which, in turn, establish a network of weak interactions initiating the formation of GR condensates (Fig. 7, ii), similarly to a model proposed elsewhere [37, 79]. We used different GR mutants to unravel the molecular determinants required for establishing this network. Particularly, since the IDRs of proteins have been proposed to participate in weak interactions of liquid condensates [13], we analyzed the capability of the GR407C mutant lacking the NTD domain to form foci. Strikingly, the mutant preserves the capability to form foci (Fig. 6a), demonstrating that GR IDR is not essential for foci formation. Indeed, the sensitivity of GR condensates to 1,7-HD treatment does not depend on this domain (Additional file 10: Supplementary Fig. S7). These observations show that other regions of GR may provide the distinctive interactions that maintain condensates. We should also mention that, despite not being essential, the NTD seems to provide GR foci sensitivity to hypertonic conditions (Fig. 6a) and thus probably stabilizes certain interactions with other molecules included in these condensates, as we discuss below.

Multivalent interactions constitute a general driving-force promoting phase separation in many known membraneless organelles [12, 48, 79]. These interactions may include IDRs as mentioned before and/or well-folded, structured regions [79, 80]. GR itself is a potential multivalent protein, since it interacts with transcriptional coregulators through two different regions (AF1 and AF2 regions in the NTD and the LBD domains, respectively) [70]. Also, GR’s ability to oligomerize results in a higher probability of establishing multivalent interactions. In this sense, we previously showed that the receptor self-association is necessary but not sufficient for foci formation [20]. The enhanced ability of the constitutive tetrameric GRtetra mutant to form foci (Fig. 6b) may rely on the higher valence of the tetramer with respect to the dimer as mentioned before. Also, the constitutive DNA-bound conformation of this mutant may favor interactions with other cofactors that also stabilize GR condensates [44, 81].

We have previously shown that GR foci also include other transcription-related molecules such as the GR coactivator NCoA-2 [20]. Here, we also found that the Mediator subunit Med1 incorporates into GR condensates (Fig. 5). Med1 acts as a GR coactivator and interacts through LXXLL motifs with the GR AF2 region in a ligand-dependent manner [82, 83]. Also, GR indirectly recruits Med1 through Med1 N-terminus [84], and the Med14 subunit interacts with the GR NTD in a ligand-independent manner [85]. The presence of many IDRs [86] and the multivalence for interactions with transcription factors and particularly with GR suggest that Mediator may promote phase separation of GR by stabilizing the interactions network within these condensates (Fig. 7, iii). Mediator is almost certainly not the only cofactor responsible for the weak intermolecular interactions within GR hubs. Many coactivators known to interact with nuclear receptors such as CBP, CARM1, and p160 family coactivators present IDRs [87–89], which may contribute to maintain liquid condensates. Also, other coactivators can interact directly or indirectly with nuclear receptors through more than one region [90, 91], thus contributing to multivalent interactions required for phase separation. For example, the coactivator NCoA-2 [20] appears to be relevant for foci formation, as the capacity to interact with NCoA-2 of different receptor conformers completely correlates with their foci forming ability [20, 28]. Since GR binds other coactivators through the same AF2 region, impairment in the interaction with NCoA-2 may coincide with diminished interactions with other coactivators, thus hindering GR phase separation. This could also explain GRmon’s inability to form foci (Fig. 6b) [20].

The hypertonic treatment increases the general concentration of biomolecules in the nucleus, due both to the reduction in the nuclear volume (Additional file 6: Supplementary Fig. S4b) and the probable exclusion of many DNA-bound molecules triggered by chromatin compaction (Fig. 2c). The higher nucleoplasmic concentrations of many of these biomolecules may shift the equilibrium towards their recruitment to previously formed GR condensates, generating the observed HOIDs. Consequently, these condensates may have a different molecular composition from the physiological GR foci and thus a different interaction network. In this context, the inability of GR407C mutant to form HOIDs (Fig. 6a) suggests that the hypertonic condensates probably include interactions between the IDR of the GR and other biomolecules.

The functional relevance of GR foci is still a matter of debate after 25 years from their initial observation [19]. At that moment, GR foci were not considered to play a role in transcription regulation since they only colocalized partially with RNA polymerase II foci and nascent RNA-enriched regions in fixed cells [19]. However, new technologies developed in the last years improved our knowledge on transcription and its regulation [92–94] making necessary to revisit these previous observations under the new theoretical background.

Particularly, it is now widely accepted that transcription occurs in bursts (reviewed in [95]) with “active” genes presenting long inactive periods and short periods of active transcription [96]. The frequency of these bursts is modulated by enhancers and promoters through mechanisms that are not completely understood [96–98]. In this context, Cho et al. [9] tracked through super-resolution microscopy the relative location of stable Mediator condensates and an actively transcribed gene locus in live cells. They found that Mediator clusters localize nearby active gene loci and only colocalize briefly with these loci. Based on their results, they proposed a dynamic “kissing” model where large Mediator clusters at enhancers transiently interact with the transcription machinery at promoters. Thus, this transient interaction may not be observed through classical methods on fixed cells with poor temporal resolution such as those used in the original GR foci studies [19].

In our work, we verified that GR condensates also include other biomolecules relevant to transcription such as the coactivator NCoA-2 [99] and the Mediator subunit Med1. In the paradigm of the “kissing model” described above, we could speculate that condensates containing GR, NCoA-2, and Med1 may only transiently interact with promoters explaining the poor colocalization observed by Van Steensel et al. [19]. Considering the stochastic nature of transcription [100], the relatively high concentration of GR, NCoA-2, Med1, and other proteins at condensates may increase the probability of transcriptionally productive interactions. In line with this proposed role, we observed a correlation between the ability of GR mutants to form condensates and their transcriptional activity. Particularly, GRmon is transcriptionally impaired [74] and is neither capable of seeding foci formation nor to be incorporated into already formed condensates (Fig. 6c). On the opposite side, the GRtetra mutant, which regulates many more genes than the wild-type receptor [75], generates more foci (Fig. 6b). Consistently, the GRN525 mutant presents low transcriptional activity [101] and does not phase separate. Nevertheless, a perfect correlation between transcriptional activity and phase separation could not be established, as the GRdim mutant, which presents impaired transcriptional activity [74], forms foci [20].

Phase separation seems to be a common response of steroid receptors to ligand activation. Indeed, it has been recently proposed that the activated estrogen receptor (ER) form liquid condensates containing clustered ER-bound enhancers and the transcriptional machinery [36, 66, 102] and that they are linked to gene activation [102]. Some other properties seem to be common to steroid receptor condensates such as the presence of Mediator [66]. However, the biochemical nature of these hubs may differ from those formed by the GR since, for example, the N-terminal IDR is essential for formation of ER foci [103] whereas it is not required for GR condensates (Fig. 6a). Nevertheless, a link between steroid receptor foci, phase separation, and transcriptional control starts to emerge, opening also relevant questions about the maturation of steroid receptor condensates during long-term hormone stimulation [36] and the possible role of steroid receptor phase separation deregulation in disease [104, 105].

## Conclusions

It is becoming clear that the distribution of transcriptional machinery in liquid condensates represents an additional layer of transcriptional control.

In this work, we dissected some of the interactions required for the formation of GR foci and showed that these structures present some properties of liquid condensates. Based on our observations, we propose that active GR molecules interact with certain chromatin regions and recruit different multivalent cofactors that interact with other molecules leading to the formation of a focus. The biological relevance of the interactions involved in GR liquid condensates suggests a role of these structures in transcriptional regulation.

Further understanding of the physical and biological properties of these condensates will likely lead to new venues for the manipulation of transcriptional output in both physiological and pathological scenarios.

## Materials and methods

### Plasmids

peGFP-C3 was acquired from Clontech. peGFP-GR, pmCherry-H2B, and pHalo-Med1 were kindly provided by Mario Galigniana (IBYME, Buenos Aires, Argentina), Robert Benezra (MSKCC, New York, USA) (Addgene plasmid #20972), and Joan Conaway (Stowers Institute, Kansas City, USA), respectively. peGFP-GR^A465T/I634A^ (GRmon) was previously described [74]. peGFP-GR407C, peGFP-GRN525, pmCherry-GR, and pmCherry-GRmon were a kind gift from Gordon Hager (NIH, Bethesda, USA).

### Cell culture and transient transfections

U2OS human osteosarcoma cells and 3617 mouse mammary adenocarcinoma cells were cultured in Dulbecco’s modified Eagle’s medium (DMEM; Thermo Fisher Scientific, Waltham, MA, USA) supplemented with 10% fetal bovine serum (FBS, Internegocios, Mercedes, Buenos Aires, Argentina) plus penicillin (100 IU/ml) and streptomycin (100 μg/ml) at 37 °C under humidified atmosphere with 4.5% CO_2_. 3617 cells were also cultured with tetracycline 5 μg/ml to maintain GFP-GR expression repressed under the Tet-off control system [43]. *Drosophila melanogaster* S2 cells were cultured in Schneider’s medium (Sigma-Aldrich) supplemented with 10% FBS at 27 °C under humidified atmosphere.

Transient transfections of U2OS cells were performed with Lipofectamine 2000 (Thermo Fisher Scientific, Waltham, MA, USA) according to manufacturer’s instructions. Briefly, 1.5 × 10^5^ cells were plated on 25-mm-diameter coverslips. The next day, the cells were transfected with 1 μg of plasmid DNA, the transfection medium was replaced with serum-free DMEM, and cells were incubated overnight with this medium prior to following treatments.

Transient transfections of S2 cells were performed with Effectene (QIAGEN, Venlo, Netherlands) according to the manufacturer’s instructions. Briefly, transfection was performed with 1.8 μg of peGFP-GR in a 24-well plate. After 48 h, the cells were placed on a 25-mm-diameter coverslip previously modified with 500 μg/ml of concanavalin A (Sigma-Aldrich, St. Louis, MO, USA).

Stable expression of GFP-GR in 3617 cells was induced by tetracycline removal 24 h prior imaging.

Cells expressing Halo-Med1 were incubated 40 min with the fluorescent dye JF549 (50 nM), which is a saturating condition for labeling Halo tagged proteins. Then the cells were washed three times for 15 min before imaging.

### Reagents and treatments

Cells were incubated with the steroid ligands in serum-free medium for at least 30 min before imaging. Dexamethasone (Dex; Sigma-Aldrich, St. Louis, MO, USA) and corticosterone (Cort, Sigma-Aldrich) were used at 10 nM and 100 nM, respectively. Cort was used in hormone removal experiments due to its lower affinity to GR, compared to Dex. Trichostatin A (TSA; Sigma-Aldrich) was used at 1 μg/ml for 16 h. 1,7-heptanediol (1,7-HD, Sigma-Aldrich) was used at 1–10% *v*/*v* for 30–180 s. Sucrose 250 mM and NaCl 100 mM were added to the culture medium and used for 1–10 min.

### Imaging

Confocal images were acquired in a FV1000 laser scanning microscope (Olympus), using an UPlanSApo 60x oil immersion objective (NA = 1.35). GFP was excited using a multi-line Ar laser at 488 nm. mCherry and JF549-labeled HaloTag were excited using a He-Ne green laser at 543 nm. The average power at the sample was 0.7 μW (U2OS cells) or 7 μW (S2 cells). Fluorescence was detected with a photomultiplier set in the pseudo photon-counting detection mode, using 500–530 nm (GFP), 560–660 nm (JF549-labeled Halo), and 600–700 nm (mCherry) filtering. Images of 256 × 256 pixels were acquired with pixel size and dwell time set at 82 nm and 10 μs, respectively. Two-channel images were acquired in sequential mode. The frame time was 0.98 s and 1.66 s for one-channel and two-channel settings, respectively.

For imaging experiments of U2OS cells expressing GR-GFP, we selected cells with mean nuclear intensities in the range 45–200 arbitrary units. This criterion guarantees images with high S/N ratio while avoiding artifacts from overexpression as further explained in the text.

For foci analyses, 10 consecutive images per cell were acquired and the averaged image was obtained for further analysis.

### Foci analysis

Individual foci were identified from averaged images obtained by confocal microscopy using the “Find Maxima” tool in ImageJ software (NIH, USA). The “noise tolerance” value (NTV) was selected according to a previous calibration. Briefly, this calibration consisted in manually selecting adequate NTVs for appropriate foci identification in images of U2OS cells expressing different levels of GFP-GR. Then, the dependence of NTV to fluorescence intensity was fitted by a lineal regression. Thereby, for every image of cell nucleus, the NTV was calculated from the mean nuclear fluorescence intensity (range 45–200). The area and mean fluorescence intensity of cell nuclei were measured from selected regions of interest (ROIs) corresponding to the nuclei. This ROI was generated by smoothing and binarizing the GFP-GR image in ImageJ. The foci density was calculated as the ratio between the number of foci found by the “Find Maxima” tool and the area of the nucleus.

To measure the foci intensity, the foci (*x*,*y*) coordinates were identified as mentioned above and then the relative foci intensity was calculated as the average intensity of a three-pixel (250 nm)-sized square centered in the focus position, normalized to the mean nuclear intensity. For two-color colocalization analyses, foci were identified in the image corresponding to one of the channels and the intensity was determined in both channels at the same coordinates.

To calculate the relative intensity of H2B-mCherry at the positions corresponding to GR foci (Int_H2B,foci_/Int_H2B,nucleus_), the foci positions were determined in the GR image as described before and the H2B-mCherry intensity was determined at the foci coordinates, relative to the mean H2B-mCherry intensity of the whole nucleus.

To analyze the shape of dots formed in hypertonic conditions, images were smoothed and binarized. The circularity (C) of structures larger than six pixels (0.04 μm^2^) was assessed in ImageJ according to Eq. 1:

1 $$ C=\frac{4\pi A}{P^2} $$

where *A* is the area of the dot and *P* is its perimeter. *C* = 1 indicates a circle while the value approaches zero for an increasingly elongated shape. To compare the circularity of dots in each condition, C histograms were built considering C dot values from many cells.

To compare foci density in S2 cells and U2OS cells with low GFP-GR expression levels, images were smoothed and binarized using a threshold equivalent to the mean nuclear intensity plus two standard deviations of the nuclear intensity. Then, structures larger than 4 pixels (0.025 μm^2^) were counted in the binary images.

### Fluorescence recovery after photobleaching

Five images were acquired before the photobleaching, then a selected nuclear region (10 × 5 μm^2^) was bleached using maximum laser power and the recovery was evaluated imaging the whole nucleus at a frame rate of 1.02 s^− 1^.

Contiguous bleached regions with and without foci from the same FRAP experiment were analyzed to compare the fluorescence recovery dynamics at the foci with respect to that at the nucleoplasm. The fluorescence intensities of each bleached region and a reference, unbleached region of the nucleus were determined for every time point and normalized to the average intensity of the same region before the photobleaching. Then, the ratio between the normalized intensity of the bleached and reference regions was calculated [106] and fitted with an empirical simple exponential equation (Eq. 2) to obtain a characteristic recovery time (*τ*_c_):

2 $$ I(t)={I}_0+{I}_R\left(1-{e}^{-\raisebox{1ex}{$t$}\!\left/ \!\raisebox{-1ex}{${\tau}_c$}\right.}\right) $$

where *I*_0_ and *I*_*R*_ are the initial and the recovered normalized intensity, respectively. Then, the characteristic recovery times obtained at a focus (*τ*_c,focus_) and a nucleoplasm region (*τ*_c,nucleoplasm_) from the same FRAP experiment were used to calculate a *τ*_c,focus_/*τ*_c,nucleoplasm_ ratio. This calculation was performed for different FRAP experiments to obtain a mean characteristic times ratio.

We did not use a theoretical model to fit the data since the dynamics of GR molecules in the nucleus is expected to be complex [20] and the purpose of the experiment was to compare the recovery in adjacent regions.

### Single particle tracking

A region of the nucleus was selected and time stacks (1000 confocal images, 128 × 128 pixels) were acquired with a pixel size set in the range of 82–97 nm and a frame rate of 3.02 s^− 1^.

To increase the signal/noise ratio, a moving average of the images’ sequence was generated using a window of 10 images. Then, single foci were individually tracked using the Globals for Images - SimFCS software (Laboratory for Fluorescence Dynamics, Irvine, CA, USA). To calculate the average drift of the nucleus during imaging, mean *x* and *y* trajectories were obtained from the foci trajectories recovered from the same image sequence and a linear regression was fitted to each mean trajectory. This drift was then subtracted from the original trajectories. The corrected trajectories were used to calculate the mean square displacement (MSD (*τ*)) according to Eq. 3:

3 $$ \mathrm{MSD}\left(\tau \right)=<{\left(x(t)-x\left(t+\tau \right)\right)}^2+{\left(y(t)-y\left(t+\tau \right)\right)}^2> $$

where *x*(*t*) and *y*(*t*) are the *x* and *y* coordinates of foci at time *t*, respectively. The mean explored distance (MED*τ* _= 100_) was calculated considering the value of MSD(*τ* = 100 s) for each trajectory and assuming that foci explored a circular area with radius equal to MED*τ*_=100_. The distance to the closest nucleolus was estimated by tracing a line between the focus center and the nucleolus border. Foci intensities were calculated from an average image of the first 50 images of the sequence and expressed as relative to the mean nuclear intensity.

### Cell nucleus volume calculations

*Z*-scans of nuclei were run using a *z*-slice of 0.5 μm. These images were used to calculate the volume of the nucleus with the Imaris software (Bitplane). A 3D image was generated and segmented, and then a mask corresponding to the segmented nucleus was selected to calculate its volume. This determination was performed for the same cells before and after the hypertonic treatment.

### Coefficient of variation (CV) analysis

The nuclear CV [21] was calculated for each nucleus as the ratio between the standard deviation and the mean nuclear H2B-mCherry fluorescence intensity.

### Statistical analysis

Most of the experiments were run at least three independent times; only a few control experiments were run twice. Results were expressed as means ± SEM from all the data obtained in the independent experiments. Statistical analyses were performed with STATISTICA 7.0 (StatSoft, Inc.) and consisted of one-way ANOVA followed by Tukey’s tests. Before statistical analysis, data were tested for homogeneity of variances using Levene’s test. If variances were not equal, a square root transformation of the data was performed and the homogeneity of variances was tested again. When this was not achieved, the statistical analysis consisted in a Kruskal-Wallis test followed by a multiple comparisons test. Student’s *t* test was performed for pairwise two-mean comparisons and to compare a mean with a value. Differences were regarded as significant at *p* < 0.05.

## Supplementary information

**Additional file 2: **Supplementary **Fig. S1.** Related to Fig. 1. (a) Representative image of a region of an U2OS cell expressing GFP-GR and incubated with Dex. Zoom-in image of a GR focus (dotted square in the left panel). (b) 3D plot showing the fluorescence intensity (represented with the indicated color code) at every *xy*-pixel of the focus image showed in A. A 2D Gaussian function (black lines) was fitted to the intensity profile (*I*) according to the following equation: $I\left(x,y\right)={I}_0+{I}_a.{e}^{-0.5{\left(\frac{x-{x}_c}{\sigma_{xy}}\right)}^2-0.5{\left(\frac{y-{y}_c}{\sigma_{xy}}\right)}^2}$. The radial waist (2*σ_xy_) was 217 + 4 nm (n_foci_ = 8), which is in the order of the optical resolution limit (~ 230 nm, [39]). Raw data can be found in Additional file 18: Supplementary Table S6.

**Additional file 3:** Supplementary **Fig. S2.** Related to Fig. 1. Representative images of U2OS cells expressing GFP-GR incubated with Dex, before and after incubation with 3 or 10% v/v 1,7-heptanediol (1,7-HD) for 30 s (Scale bar: 5 μm).

**Additional file 4: **Supplementary **Fig. S3.** Related to Fig. 1. (a) *(Left)* Representative images of 3617 cells stably expressing GFP-GR and incubated with Dex, before and after incubation with 1% v/v 1,7-heptanediol (1,7-HD) for 30 s. Scale bar: 5 μm. The zoomed image corresponds to the region indicated by the dashed square. (*Right*) Mean foci density after 1,7-HD incubation relative to the foci density in the same cells before 1,7-HD incubation (n_cells_ = 7). The asterisk (*) denotes a relative foci density significantly different from 1 (*p* < 0.05). Raw data can be found in Additional file 19: Supplementary Table S7. (b) 3617 cells stably expressing GFP-GR and U2OS transiently expressing GFP-GR were incubated with Dex and imaged by confocal microscopy. Z-stacks of images of 212 μm-sized fields were acquired to sample 3617 cells (*n* = 117) and U2OS cells (*n* = 85) and the intensity of each nucleus was calculated at its mean plane. Histograms of GFP-GR nuclear intensities for 3617 (dark green line) and U2OS (light green line) cells. The gray band shows the intensity range (45–200) used to select U2OS cells in our work. The intensity levels of these cells were similar to those of 3617 cells stably expressing GFP-GR. Raw data can be found in Additional file 19: Supplementary Table S7 (c) *(Left)* Representative images of 3617 cells stably expressing GFP-GR incubated with Dex, before and after incubation with medium supplemented with NaCl 100 mM for 1 min. Scale bar: 5 μm. The zoomed image corresponds to the region indicated by the dashed square. (*Right*) Mean foci density in cells incubated with Dex (n_cells_ = 8) and in cells incubated with Dex and medium supplemented with NaCl (n_cells_ = 6). The asterisk (*) denotes a significantly different foci density (*p* < 0.05) respect to that obtained in isotonic medium. Raw data can be found in Additional file 19: Supplementary Table S7.

**Additional file 6: **Supplementary **Fig. S4.** Related to Fig. 2. (a) U2OS cells expressing GFP-GR were incubated with Dex, then with medium supplemented with 250 mM sucrose for 1 min and imaged by confocal microscopy. Representative images of the same cells before and after sucrose incubation, and after re-introducing the cells in isotonic medium (Sucrose washout) (Scale bar: 5 μm). (b) Representative images of U2OS cells expressing GFP-GR incubated with Dex and then with medium supplemented with 100 mM NaCl for 1 min (Scale bar: 5 μm). (*Left, bottom panels*) *yz* images of the same cell before and after NaCl incubation at the plane indicated with a gray dashed line. (*Right panel*) Changes in the volume and the GR intensity of the nucleus after NaCl incubation are represented relative to the values measured in isotonic medium. The asterisk (*) denotes a significantly different value from 1 (*p* < 0.05) (n_cells_ = 6). Raw data can be found in Additional file 20: Supplementary Table S8. (c) Representative images of regions of U2OS cells co-expressing H2B-mCherry and GFP-GR or GFP alone incubated with vehicle (Veh) or Dex and then with medium supplemented with 100 mM NaCl for 1 min (Scale bar: 2 μm). GFP images were binarized to obtain a dots mask and the dots circularity was calculated according to Eq. 1. The histogram shows the circularity distribution for each condition (GR + Dex: n_dots_ = 670; n_cells_ = 6; GR + Veh: n_dots_ = 202; n_cells_ = 4; GFP: n_dots_ = 285; n_cells_ = 4). Raw data can be found in Additional file 20: Supplementary Table S8.

**Additional file 8: **Supplementary **Fig. S5.** Related to Fig. 3. U2OS cells expressing GFP-GR were incubated with Dex and imaged as a function of time. These movies were analyzed using the single-particle tracking routine to obtain 2D trajectories of GR foci. The mean square displacement (MSD) was calculated as a function of the time lag (Eq. 3, see “Materials and methods”) and the mean explored distance was calculated from the MSD values at time lag =100 s. Foci intensities were calculated from an average image of the first 50 images of the sequence and expressed relative to the mean nuclear intensity. (*Left*) The mean explored distance is represented as a function of foci intensity, discriminating populations according to their distance to nucleoli. Proximal (red dots, n_foci_ = 30) or distal (blue dots, n_foci_ = 50) foci were classified according to their distance to nucleoli using a threshold of 0.5 μm. (*Middle*) Mean foci intensity of proximal and distal foci. The asterisk (*) denotes significantly different foci intensities (p < 0.05). (*Right*) Mean explored distance of dimmer distal foci (foci intensity < 1.8, n_foci_ = 40) and brighter distal foci (foci intensity > 1.8, n_foci_ = 10). The asterisk (*) denotes significantly different explored distances (p < 0.05). Raw data can be found in Additional file 16: Supplementary Table S4.

**Additional file 9:** Supplementary **Fig. S6.** Related to Fig. 5. U2OS cells co-expressing GFP-GR and JF549-labeled Halo-Med1 were incubated with vehicle (Veh) or Dex, then with medium supplemented with 100 mM NaCl (when indicated) and imaged by confocal microscopy. The dot plots represent the GFP-GR intensity as a function of the JF549 intensity at each Med1 condensate previously identified from the JF549 image. The intensity values were normalized to the mean nuclear intensity of each cell, in each channel (Veh: n_foci_ = 138; n_cells_ = 8; Dex: n_foci_ = 358; n_cells_ = 13; Dex + NaCl: n_foci_ = 445; n_cells_ = 5). Raw data can be found in Additional file 21: Supplementary Table S9.

**Additional file 10: **Supplementary **Fig. S7.** Related to Fig. 6. Representative images of U2OS cells expressing GFP-GR or GFP-GR407C incubated with Dex, before and after incubation with 1% v/v 1,7-heptanediol (1,7-HD) for 30 s (Scale bar: 5 μm). The zoomed images correspond to the regions indicated by the dashed squares (Scale bar: 2 μm). (*right*) Mean foci density in cells expressing GFP-GR or GFP-GR407C incubated with Dex and treated with 1,7-HD 1% (n_cells,GR + Dex_ = 146; n_cells,GR + Dex + 1,7-HD_ = 20; n_cells,GR407C + Dex_ = 43; n_cells,GR407C + Dex + 1,7-HD_ = 8). The asterisk (*) denotes a significantly different foci density (p < 0.05) with respect to that obtained for the same GR variant without 1,7-HD. Raw data can be found in Additional file 22: Supplementary Table S10.

**Additional file 11: **Supplementary **Fig. S8.** Related to Fig. 6. U2OS cells expressing GFP fused to wild type GR, GRtetra, GR407C, GRN525 or GRmon were incubated with Dex and imaged by confocal microscopy. Foci analysis was performed as described in “Materials and methods.” Cumulative histograms of foci intensities (relative to the mean nuclear intensity) for each condition (GR: n_foci_ = 879; n_cells_ = 10; GRtetra: n_foci_ = 1151; n_cells_ = 8; GR407C: n_foci_ = 1047; n_cells_ = 9; GRN525: n_foci_ = 128; n_cells_ = 9; GRmon: n_foci_ = 96; n_cells_ = 8). The foci counts were normalized to the total number of foci analyzed. The mean foci intensities (± SEM) for each GR variant were: GR: 1.343 ± 0.008; GRtetra: 1.368 ± 0.009; GR407C: 1.332 ± 0.008; GRN525: 1.199 ± 0.013; GRmon: 1.172 ± 0.025). Raw data can be found in Additional file 23: Supplementary Table S11.

**Additional file 12: **Supplementary **Fig. S9.** Related to Fig. 6. U2OS cells co-expressing GFP-GR and mCherry-GR, GFP-GR and mCherry-GRmon or mCherry-GR and GFP-GRmon were incubated with Dex and imaged by confocal microscopy. GR condensates were located as described in “Materials and methods” and their intensity in the green and red images were determined and normalized to the mean nuclear intensity of each cell, in each channel. The dot plots represent (*left*) the mCherry-GR intensity as a function of GFP-GR intensity at each condensate previously identified from the green image (n_foci_ = 365; n_cells_ = 5) and (*right*) the mCherry-GRmon (red dots, n_foci_ = 416; n_cells_ = 6) or GFP-GRmon (orange dots, n_foci_ = 165; n_cells_ = 5) intensity as a function of GFP-GR or mCherry-GR intensity, respectively, at each GR condensate. Raw data can be found in Additional file 24: Supplementary Table S12.

**Additional file 13:** Supplementary **Table S1.** Related to Fig. 1. Raw data.

**Additional file 14:** Supplementary **Table S2.** Related to Fig. 2. Raw data.

**Additional file 15:** Supplementary **Table S3.** Related to Fig. 3 and Supplementary Fig. S5. Raw data.

**Additional file 16:** Supplementary **Table S4.** Related to Fig. 4. Raw data.

**Additional file 17:** Supplementary **Table S5.** Related to Fig. 6. Raw data.

**Additional file 18:** Supplementary **Table S6.** Related to Supplementary Fig. S1. Raw data.

**Additional file 19:** Supplementary **Table S7.** Related to Supplementary Fig. S3. Raw data.

**Additional file 20:** Supplementary **Table S8.** Related to Supplementary Fig. S4. Raw data.

**Additional file 21:** Supplementary **Table S9.** Related to Supplementary Fig. S6. Raw data.

**Additional file 22:** Supplementary **Table S10.** Related to Supplementary Fig. S7. Raw data.

**Additional file 23:** Supplementary **Table S11.** Related to Supplementary Fig. S8. Raw data.

**Additional file 24:** Supplementary **Table S12.** Related to Supplementary Fig. S9. Raw data.

## Data Availability

All data generated or analyzed during this study are included in this published article and its supplementary information files. Raw data can be found in Additional files 13, 14, 15, 16, 17, 18, 19, 20, 21, 22, 23, and 24 (Supplementary Tables S1-S12).

## Abbreviations

1,7-HD: 1,7-Heptanediol
Cort: Corticosterone
Dex: Dexamethasone
GR: Glucocorticoid receptor
HOID: Hyperosmotic-induced dot
IDR: Intrinsically disordered region
LBD: Ligand-binding domain
MSD: Mean square displacement
NTD: N-terminal domain
TSA: Trichostatin A
Veh: Vehicle
