# Supplementary figures and images for: Unraveling the molecular interactions involved in phase separation of glucocorticoid receptor
Source: BMC Biol. 2020 Jun 2;18:59. doi: 10.1186/s12915-020-00788-2 (PMC7268505; doi:10.1186/s12915-020-00788-2)

**Supplementary Fig. S1**

**a**

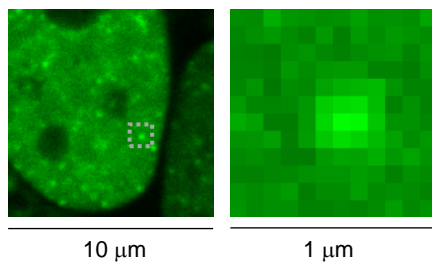

**b**

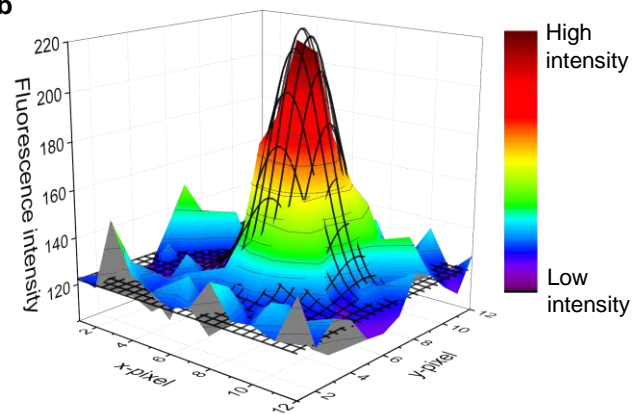

Supplement: Supplementary file 4 — Additional file 2: Supplementary Fig. S1. Related to Fig. 1. (a) Representative image of a region of an U2OS cell expressing GFP-GR and incubated with Dex. Zoom-in image of a GR focus (dotted square in the left panel). (b) 3D plot showing the fluorescence intensity (represented with the indicated color code) at every xy-pixel of the focus image showed in A. A 2D Gaussian function (black lines) was fitted to the intensity profile (I) according to the following equation: $I\left(x,y\right)={I}_0+{I}_a.{e}^{-0.5{\left(\frac{x-{x}_c}{\sigma_{xy}}\right)}^2-0.5{\left(\frac{y-{y}_c}{\sigma_{xy}}\right)}^2}$Ixy=I0+Ia.e−0.5x−xcσxy2−0.5y−ycσxy2. The radial waist (2*σxy) was 217 + 4 nm (nfoci = 8), which is in the order of the optical resolution limit (~ 230 nm, [39]). Raw data can be found in Additional file 18: Supplementary Table S6. [file 12915_2020_788_MOESM2_ESM.pdf]

**Supplementary Fig. S2**

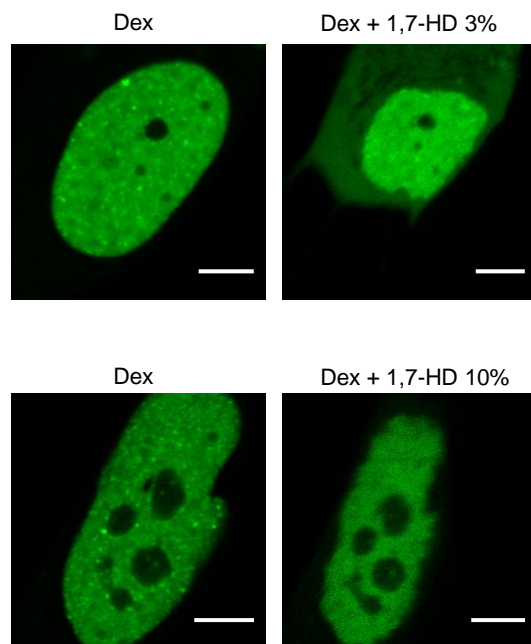

Supplement: Supplementary file 5 — Additional file 3: Supplementary Fig. S2. Related to Fig. 1. Representative images of U2OS cells expressing GFP-GR incubated with Dex, before and after incubation with 3 or 10% v/v 1,7-heptanediol (1,7-HD) for 30 s (Scale bar: 5 μm). [file 12915_2020_788_MOESM3_ESM.pdf]

# Supplementary Fig. S3

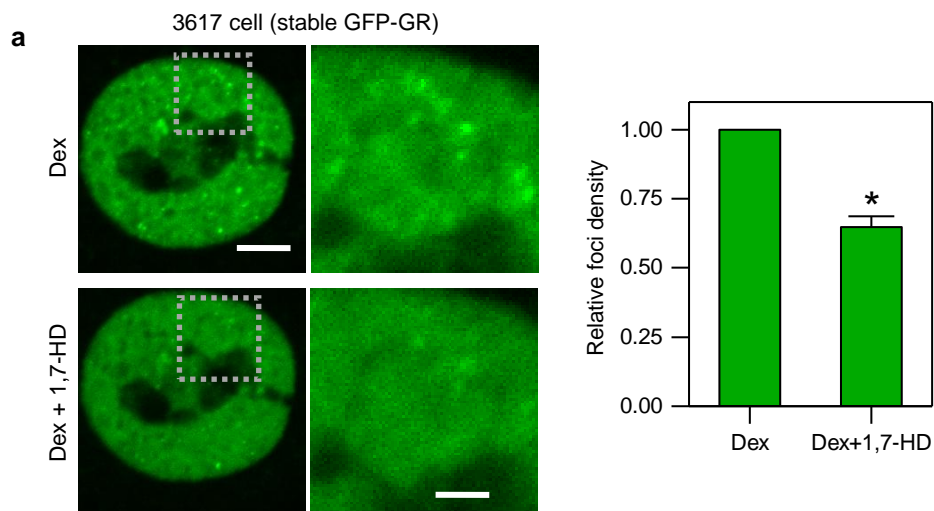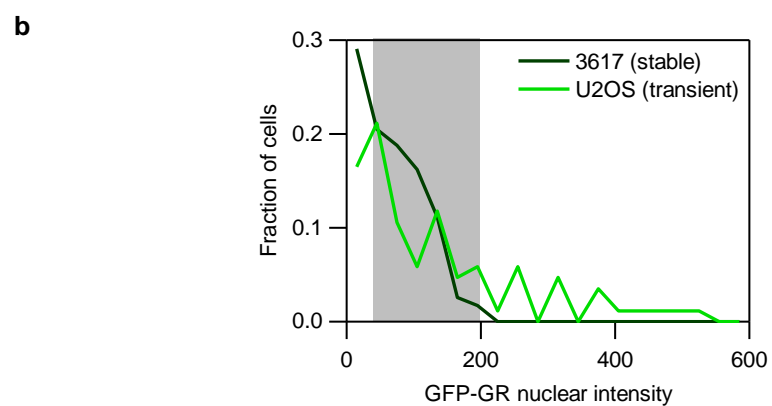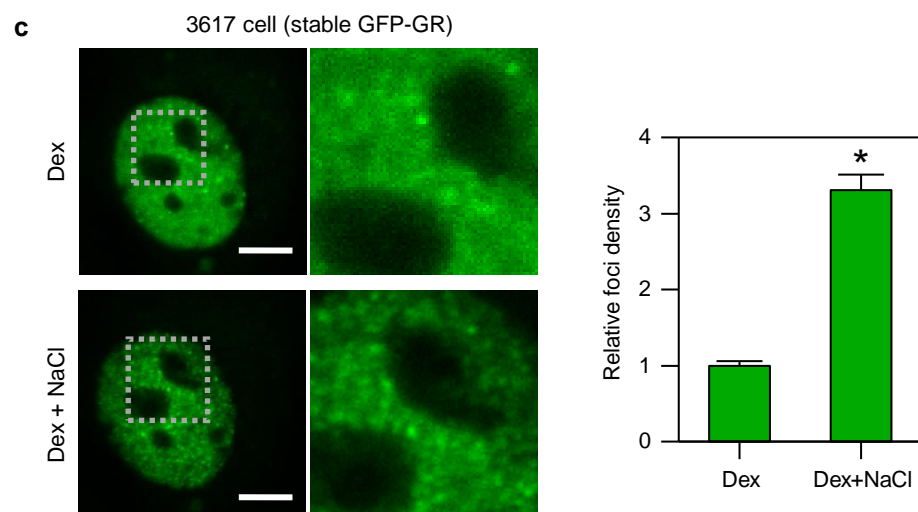

Supplement: Supplementary file 6 — Additional file 4: Supplementary Fig. S3. Related to Fig. 1. (a) (Left) Representative images of 3617 cells stably expressing GFP-GR and incubated with Dex, before and after incubation with 1% v/v 1,7-heptanediol (1,7-HD) for 30 s. Scale bar: 5 μm. The zoomed image corresponds to the region indicated by the dashed square. (Right) Mean foci density after 1,7-HD incubation relative to the foci density in the same cells before 1,7-HD incubation (ncells = 7). The asterisk (*) denotes a relative foci density significantly different from 1 (p < 0.05). Raw data can be found in Additional file 19: Supplementary Table S7. (b) 3617 cells stably expressing GFP-GR and U2OS transiently expressing GFP-GR were incubated with Dex and imaged by confocal microscopy. Z-stacks of images of 212 μm-sized fields were acquired to sample 3617 cells (n = 117) and U2OS cells (n = 85) and the intensity of each nucleus was calculated at its mean plane. Histograms of GFP-GR nuclear intensities for 3617 (dark green line) and U2OS (light green line) cells. The gray band shows the intensity range (45–200) used to select U2OS cells in our work. The intensity levels of these cells were similar to those of 3617 cells stably expressing GFP-GR. Raw data can be found in Additional file 19: Supplementary Table S7 (c) (Left) Representative images of 3617 cells stably expressing GFP-GR incubated with Dex, before and after incubation with medium supplemented with NaCl 100 mM for 1 min. Scale bar: 5 μm. The zoomed image corresponds to the region indicated by the dashed square. (Right) Mean foci density in cells incubated with Dex (ncells = 8) and in cells incubated with Dex and medium supplemented with NaCl (ncells = 6). The asterisk (*) denotes a significantly different foci density (p < 0.05) respect to that obtained in isotonic medium. Raw data can be found in Additional file 19: Supplementary Table S7. [file 12915_2020_788_MOESM4_ESM.pdf]

**Supplementary Fig. S4**

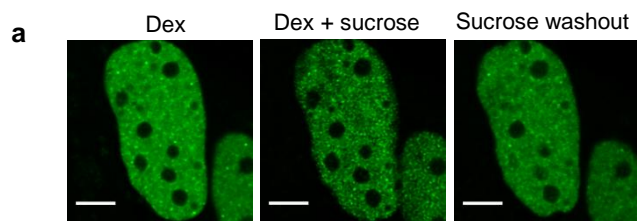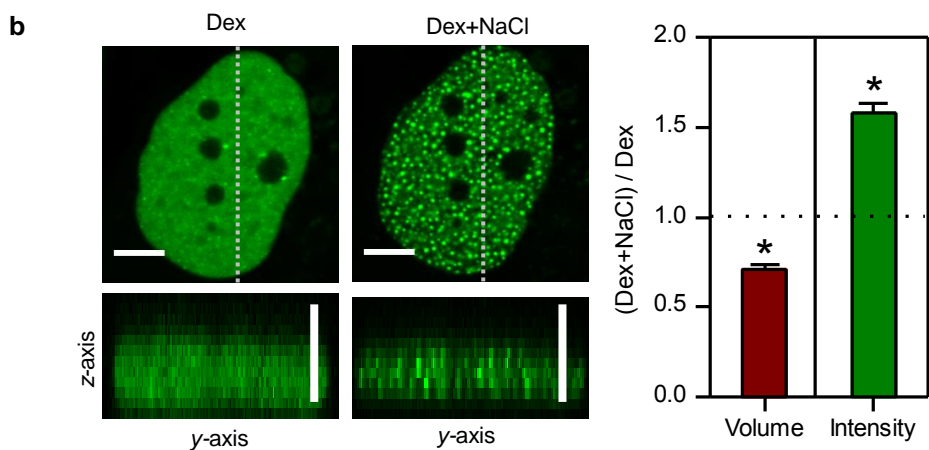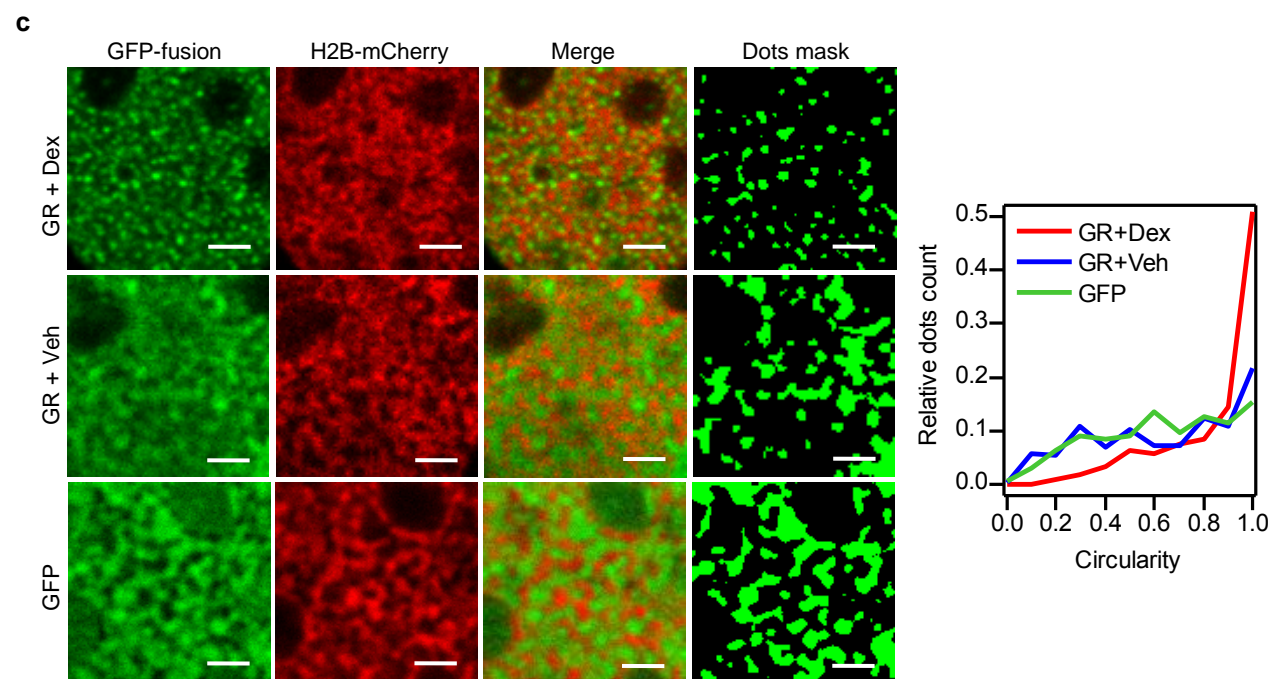

Supplement: Supplementary file 7 — Additional file 6: Supplementary Fig. S4. Related to Fig. 2. (a) U2OS cells expressing GFP-GR were incubated with Dex, then with medium supplemented with 250 mM sucrose for 1 min and imaged by confocal microscopy. Representative images of the same cells before and after sucrose incubation, and after re-introducing the cells in isotonic medium (Sucrose washout) (Scale bar: 5 μm). (b) Representative images of U2OS cells expressing GFP-GR incubated with Dex and then with medium supplemented with 100 mM NaCl for 1 min (Scale bar: 5 μm). (Left, bottom panels) yz images of the same cell before and after NaCl incubation at the plane indicated with a gray dashed line. (Right panel) Changes in the volume and the GR intensity of the nucleus after NaCl incubation are represented relative to the values measured in isotonic medium. The asterisk (*) denotes a significantly different value from 1 (p < 0.05) (ncells = 6). Raw data can be found in Additional file 20: Supplementary Table S8. (c) Representative images of regions of U2OS cells co-expressing H2B-mCherry and GFP-GR or GFP alone incubated with vehicle (Veh) or Dex and then with medium supplemented with 100 mM NaCl for 1 min (Scale bar: 2 μm). GFP images were binarized to obtain a dots mask and the dots circularity was calculated according to Eq. 1. The histogram shows the circularity distribution for each condition (GR + Dex: ndots = 670; ncells = 6; GR + Veh: ndots = 202; ncells = 4; GFP: ndots = 285; ncells = 4). Raw data can be found in Additional file 20: Supplementary Table S8. [file 12915_2020_788_MOESM6_ESM.pdf]

**Supplementary Fig. S5**

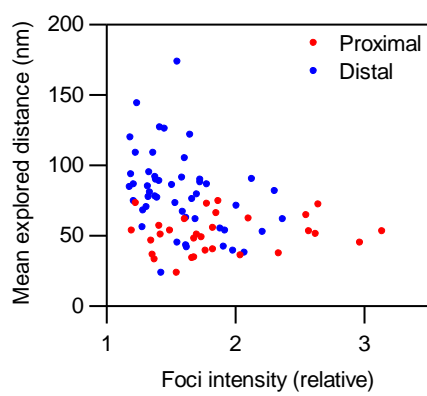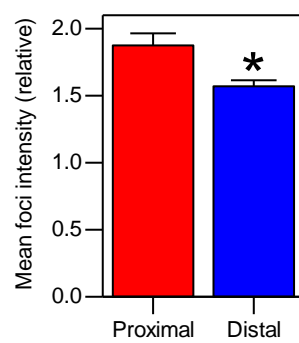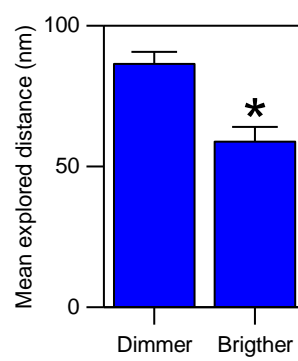

Supplement: Supplementary file 8 — Additional file 8: Supplementary Fig. S5. Related to Fig. 3. U2OS cells expressing GFP-GR were incubated with Dex and imaged as a function of time. These movies were analyzed using the single-particle tracking routine to obtain 2D trajectories of GR foci. The mean square displacement (MSD) was calculated as a function of the time lag (Eq. 3, see “Materials and methods”) and the mean explored distance was calculated from the MSD values at time lag =100 s. Foci intensities were calculated from an average image of the first 50 images of the sequence and expressed relative to the mean nuclear intensity. (Left) The mean explored distance is represented as a function of foci intensity, discriminating populations according to their distance to nucleoli. Proximal (red dots, nfoci = 30) or distal (blue dots, nfoci = 50) foci were classified according to their distance to nucleoli using a threshold of 0.5 μm. (Middle) Mean foci intensity of proximal and distal foci. The asterisk (*) denotes significantly different foci intensities (p < 0.05). (Right) Mean explored distance of dimmer distal foci (foci intensity < 1.8, nfoci = 40) and brighter distal foci (foci intensity > 1.8, nfoci = 10). The asterisk (*) denotes significantly different explored distances (p < 0.05). Raw data can be found in Additional file 16: Supplementary Table S4. [file 12915_2020_788_MOESM8_ESM.pdf]

**Supplementary Fig. S6**

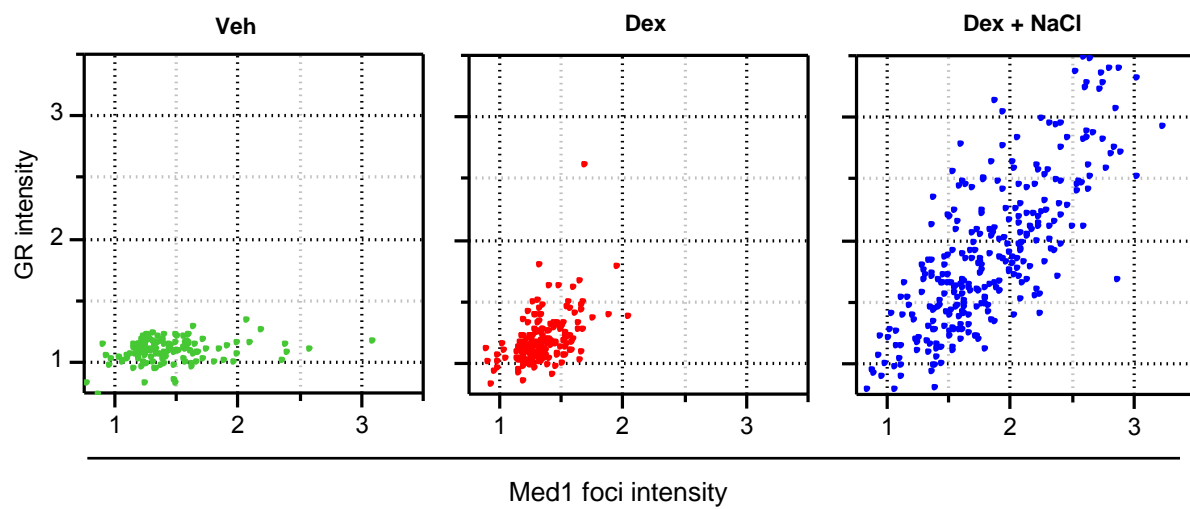

Supplement: Supplementary file 9 — Additional file 9: Supplementary Fig. S6. Related to Fig. 5. U2OS cells co-expressing GFP-GR and JF549-labeled Halo-Med1 were incubated with vehicle (Veh) or Dex, then with medium supplemented with 100 mM NaCl (when indicated) and imaged by confocal microscopy. The dot plots represent the GFP-GR intensity as a function of the JF549 intensity at each Med1 condensate previously identified from the JF549 image. The intensity values were normalized to the mean nuclear intensity of each cell, in each channel (Veh: nfoci = 138; ncells = 8; Dex: nfoci = 358; ncells = 13; Dex + NaCl: nfoci = 445; ncells = 5). Raw data can be found in Additional file 21: Supplementary Table S9. [file 12915_2020_788_MOESM9_ESM.pdf]

**Supplementary Fig. S7**

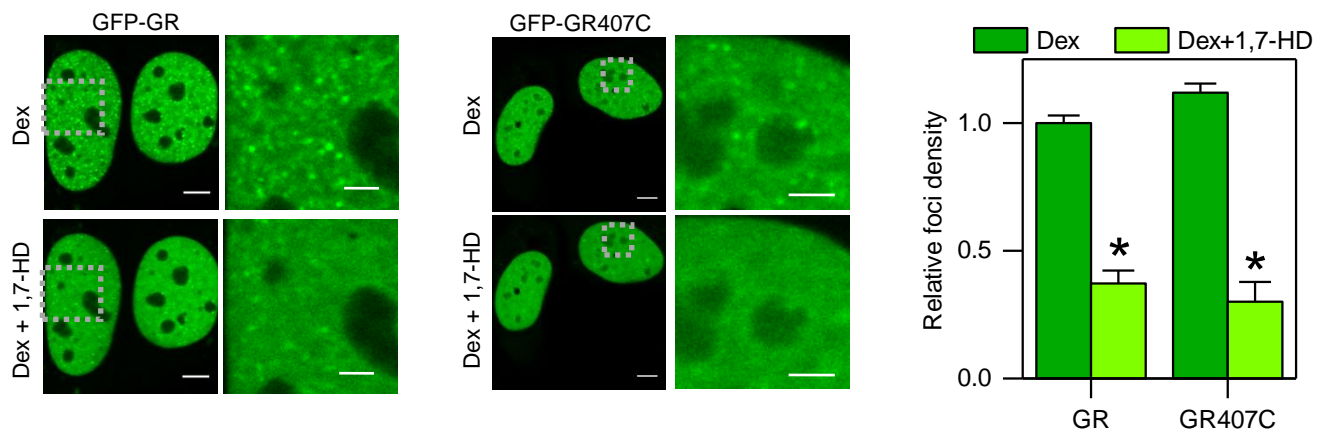

Supplement: Supplementary file 10 — Additional file 10: Supplementary Fig. S7. Related to Fig. 6. Representative images of U2OS cells expressing GFP-GR or GFP-GR407C incubated with Dex, before and after incubation with 1% v/v 1,7-heptanediol (1,7-HD) for 30 s (Scale bar: 5 μm). The zoomed images correspond to the regions indicated by the dashed squares (Scale bar: 2 μm). (right) Mean foci density in cells expressing GFP-GR or GFP-GR407C incubated with Dex and treated with 1,7-HD 1% (ncells,GR + Dex = 146; ncells,GR + Dex + 1,7-HD = 20; ncells,GR407C + Dex = 43; ncells,GR407C + Dex + 1,7-HD = 8). The asterisk (*) denotes a significantly different foci density (p < 0.05) with respect to that obtained for the same GR variant without 1,7-HD. Raw data can be found in Additional file 22: Supplementary Table S10. [file 12915_2020_788_MOESM10_ESM.pdf]

**Supplementary Fig. S8**

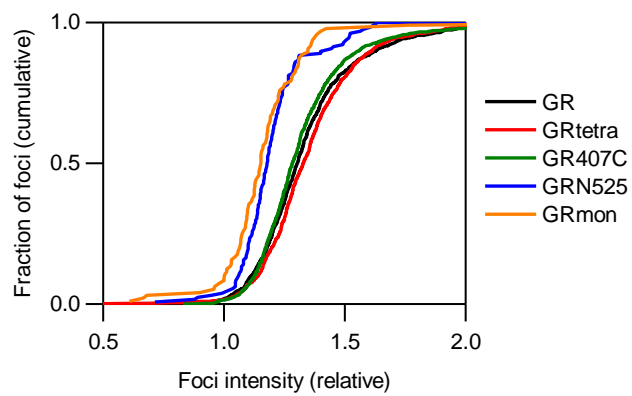

Supplement: Supplementary file 11 — Additional file 11: Supplementary Fig. S8. Related to Fig. 6. U2OS cells expressing GFP fused to wild type GR, GRtetra, GR407C, GRN525 or GRmon were incubated with Dex and imaged by confocal microscopy. Foci analysis was performed as described in “Materials and methods.” Cumulative histograms of foci intensities (relative to the mean nuclear intensity) for each condition (GR: nfoci = 879; ncells = 10; GRtetra: nfoci = 1151; ncells = 8; GR407C: nfoci = 1047; ncells = 9; GRN525: nfoci = 128; ncells = 9; GRmon: nfoci = 96; ncells = 8). The foci counts were normalized to the total number of foci analyzed. The mean foci intensities (± SEM) for each GR variant were: GR: 1.343 ± 0.008; GRtetra: 1.368 ± 0.009; GR407C: 1.332 ± 0.008; GRN525: 1.199 ± 0.013; GRmon: 1.172 ± 0.025). Raw data can be found in Additional file 23: Supplementary Table S11. [file 12915_2020_788_MOESM11_ESM.pdf]

Supplementary Fig. S9

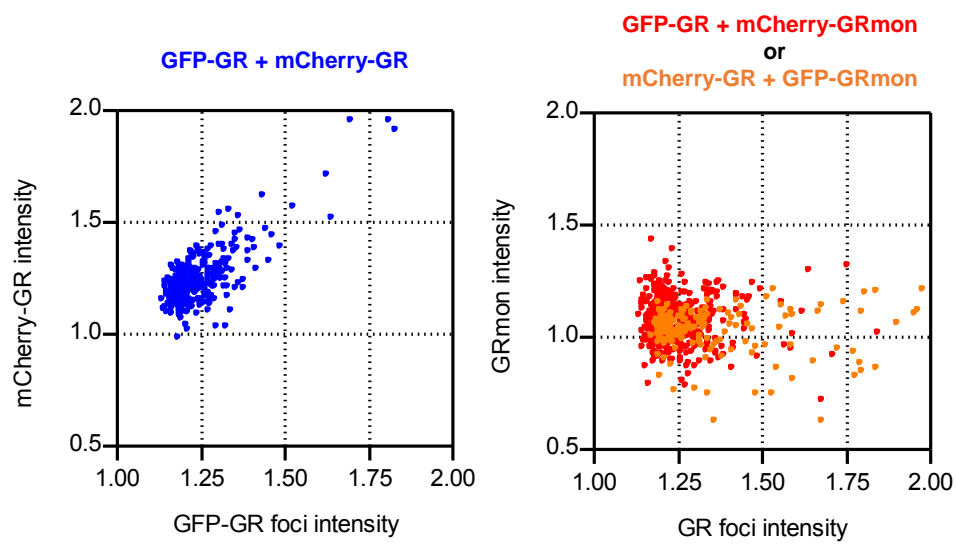

Supplement: Supplementary file 12 — Additional file 12: Supplementary Fig. S9. Related to Fig. 6. U2OS cells co-expressing GFP-GR and mCherry-GR, GFP-GR and mCherry-GRmon or mCherry-GR and GFP-GRmon were incubated with Dex and imaged by confocal microscopy. GR condensates were located as described in “Materials and methods” and their intensity in the green and red images were determined and normalized to the mean nuclear intensity of each cell, in each channel. The dot plots represent (left) the mCherry-GR intensity as a function of GFP-GR intensity at each condensate previously identified from the green image (nfoci = 365; ncells = 5) and (right) the mCherry-GRmon (red dots, nfoci = 416; ncells = 6) or GFP-GRmon (orange dots, nfoci = 165; ncells = 5) intensity as a function of GFP-GR or mCherry-GR intensity, respectively, at each GR condensate. Raw data can be found in Additional file 24: Supplementary Table S12. [file 12915_2020_788_MOESM12_ESM.pdf]
